# Structural diversity and substrate preferences of three tannase enzymes encoded by the anaerobic bacterium *Clostridium butyricum*

**DOI:** 10.1016/j.jbc.2022.101758

**Published:** 2022-02-21

**Authors:** Amanda Sörensen Ristinmaa, Tom Coleman, Leona Cesar, Annika Langborg Weinmann, Scott Mazurkewich, Gisela Brändén, Merima Hasani, Johan Larsbrink

**Affiliations:** 1 Division of Industrial Biotechnology, Department of Biology and Biological Engineering, Chalmers University of Technology, Gothenburg, Sweden; 2 Early Chemical Development, Pharmaceutical Sciences, AstraZeneca, Gothenburg, Sweden; 3 Wallenberg Wood Science Center, Chalmers University of Technology, Gothenburg, Sweden; 4 Department of Chemistry and Molecular Biology, University of Gothenburg, Gothenburg, Sweden; 5 Division of Forest Products and Chemical Engineering, Department of Chemistry and Chemical Engineering, Chalmers University of Technology, Gothenburg, Sweden

**Keywords:** serine esterase, enzyme kinetics, crystal structure, enzyme structure, high-performance liquid chromatography (HPLC), tannase, tannin, oak bark, extractives, tannin acyl hydrolase, ACN, acetonitrile, BLAST, Basic Local Alignment Search Tool, GG, β-Glucogallin, HG, hexyl gallate, HPAEC-PAD, high-performance anion exchange chromatography with pulsed amperometric detection, HPLC-PDA, high-performance liquid chromatography with photo diode array detection, MG, methyl gallate, PG, propyl gallate, SDS-PAGE, sodium dodecyl sulfate–polyacrylamide gel electrophoresis

## Abstract

Tannins are secondary metabolites that are enriched in the bark, roots, and knots in trees and are known to hinder microbial attack. The biological degradation of water-soluble gallotannins, such as tannic acid, is initiated by tannase enzymes (EC 3.1.1.20), which are esterases able to liberate gallic acid from aromatic-sugar complexes. However, only few tannases have previously been studied in detail. Here, for the first time, we biochemically and structurally characterize three tannases from a single organism, the anaerobic bacterium *Clostridium butyricum*, which inhabits both soil and gut environments. The enzymes were named *Cb*Tan1-3, and we show that each one exhibits a unique substrate preference on a range of galloyl ester model substrates; *Cb*Tan1 and 3 demonstrated preference toward galloyl esters linked to glucose, while *Cb*Tan2 was more promiscuous. All enzymes were also active on oak bark extractives. Furthermore, we solved the crystal structure of *Cb*Tan2 and produced homology models for *Cb*Tan1 and 3. In each structure, the catalytic triad and gallate-binding regions in the core domain were found in very similar positions in the active site compared with other bacterial tannases, suggesting a similar mechanism of action among these enzymes, though large inserts in each enzyme showcase overall structural diversity. In conclusion, the varied structural features and substrate specificities of the *C. butyricum* tannases indicate that they have different biological roles and could further be used in development of new valorization strategies for renewable plant biomass.

Bark forms the outer layer of trunks and branches of trees, and as such, it has an important role in the defense against both abiotic and biotic stresses. Like wood, bark is composed of a heterogeneous matrix of cellulose, hemicellulose, and lignin, but it has a much higher content of so-called extractive compounds (extractives) (1). The extractives are secondary metabolites composed of both hydrophilic and hydrophobic molecules and can, as the name suggests, be extracted from biomass using different polar and nonpolar solvents. Tannins are one of the main components of the hydrophilic bark extractives and are composed of a range of high-molecular-weight compounds (1, 2). Tannins aid in protecting plants against microbial attack by binding to and precipitating microbial proteins (3), which in turn inhibits the growth of various microorganisms (4).

Tannins are divided into four subgroups: gallotannins, ellagitannins, condensed tannins, and complex tannins, based on their structural properties (Fig. 1) (2). Gallotannins and ellagitannins are classified as hydrolysable tannins and are composed of either a galloyl or ellagoyl moiety linked to a glucose moiety. Condensed tannins are composed of catechin units coupled together by carbon–carbon bonds, and complex tannins contain either a gallotannin or an ellagitannin linked to a catechin unit. Generally, hardwood trees, such as oak, have higher concentrations of tannins compared with softwoods and a higher concentration of hydrolysable tannins than condensed tannins (5). Oak bark has historically been used as a source to extract tannins for tanning leather, and tannins can represent 20% of the dry weight of the bark (2). The type and amount of tannin extracted from the oak bark depend on the season, the oak species, and type of solvent used (6).

**Figure 1:**
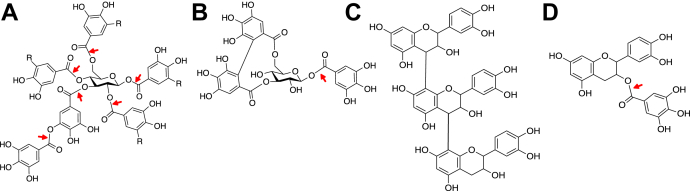
**Tannin subgroups and representative structures. Ester bonds that are hydrolysable by tannases are marked with red arrows.** Water-soluble oak bark extractives are composed of all these subgroups at different concentrations, but predominantly gallotannins and ellagitannins. The two groups of hydrolysable tannins are *A*) gallotannins (hexagalloylglucose (3-*O*-galloyl-1,2,4,6-tetra-*O*-galloylglucose) or tannic acid if R=gallate), and *B*) ellagitannins (corilagin). *C*) Condensed tannins (procyanidin C1), and *D*) complex tannins (catechin/epicatechin gallate). Tannases have not been shown to be active on the condensed tannin structures or catalyze the cleavage of the ester bonds between ellagic acid and the *O*3 and *O*6 positions of glucose in ellagitannin.

Despite the antimicrobial activity of tannin, there are microorganisms isolated from both gut and soil environments that can degrade and/or utilize tannins as a carbon source. The initial hydrolysis of tannins is performed by tannases (tannin acyl hydrolases, EC 3.1.1.20), which catalyze the hydrolysis of ester and depside linkages in tannins to release, *e.g.*, gallic acid, catechin, and glucose (7, 8, 9). Tannases have been used in hydrolysis of gallotannins or more complex natural sources such as green tea, which leads to the release of gallic acid and epigallocatechin (10). Even though many microorganisms able to use tannin as a carbon source have been found, only a limited number of tannases have been recombinantly produced, and even fewer have had their kinetic parameters determined (11, 12, 13).

Tannases have been identified from bacteria, fungi, yeast, and plants (14, 15, 16, 17). Fungal tannases have been classified within the feruloyl esterase (E.C. 3.1.1.73) subclass of carboxylic acid esterases, though only few fungal tannases have been characterized, and these include enzymes able to cleave both tannase and feruloyl esterase model substrates (15). Interestingly, the bacterial and the fungal tannases are not closely related based on structural alignment in the ESTHER database, a repository of esterase and α/β hydrolases (18). Bacterial tannases have been proposed to be divided into two groups, subtypes A and B, based respectively on the absence or presence of an aspartate residue in the catalytic triad, absence or presence of a signal peptide, and the protein length, with subtype A tannases being around 600 residues, and subtype B being somewhat smaller (∼470–570 residues) (16, 19). No significant differences in the biochemical properties between the two proposed subgroups have however been demonstrated, apart from a few type B tannases having been shown to preferentially cleave gallate esters with longer alkyl chains (20, 21). Furthermore, there are tannases categorized as subtype B that contain signal peptides in contrast to the subtype definition (16, 19), suggesting that this may not be a reliable indicator.

Structural information on tannases is very sparse, and only two bacterial tannases have to date been structurally determined using X-ray crystallography, from the species *Lactiplantibacillus plantarum* (formerly *Lactobacillus plantarum*) and *Fusobacterium nucleatum* (22, 23). Both enzymes adopt an α/β hydrolase fold, though this core fold structure accounts for only around half the total residues in the enzymes due to multiple inserts (23). Notably, there is an antiparallel-β-sheet cap structure covering the active site in the solved structures. Ligand structures of the *L. plantarum* enzyme in complex with a gallate molecule revealed the position of the active site and how the carboxylate group on the ligand interacts with the Ser and His residues of the catalytic triad, and furthermore, how the hydroxy groups of the gallate are anchored in place *via* Lys, Asp, and Glu residues (23). Galloyl esters with varied alkyl chains (methyl, ethyl, dodecyl) were also cocrystallized into inactive Ser→Ala enzyme variants, though electron density could only be observed for two carbon atoms of the dodecyl chain, suggesting that long aliphatic regions are flexible in solution and do not interact strongly with this enzyme. To date, only two microorganisms have been reported to encode two tannases: the bacteria *L. plantarum* and *Streptococcus gallolyticus* (12, 20, 24, 25). Consequently, there is a lack of both knowledge and comparative studies on the multiplicity of tannases encoded by single organisms and whether these have different biological roles.

In the present study, we have investigated three putative tannases encoded by the anaerobic bacterium *Clostridium butyricum*, initially isolated from the human gut but which has also been found in soil (26). There are no reports on tannin utilization by *C. butyricum*, though a gallate decarboxylase converting gallic acid into pyrogallol and carbon dioxide has been identified (27). The reason for *C. butyricum* encoding multiple tannases is thus not known but could reflect an ability to either metabolize or detoxify tannins to support growth in diverse environments. The three enzymes were shown to be phylogenetically dissimilar, and their activities were initially characterized on model substrates, for which they displayed distinct biochemical properties. The enzymes were further shown to be active on oak bark tannins, which has not previously been demonstrated for tannases. Furthermore, we solved the structure of one of the enzymes (*Cb*Tan2) using X-ray crystallography and have modeled the structures of the other two enzymes (*Cb*Tan1 and 3) for comparative analyses. We show that the *C. butyricum* tannases possess highly conserved active sites, but also diverse insertions that may reflect distinct biological roles.

## Results

### Phylogenetic and sequence analysis

**Figure 2:**
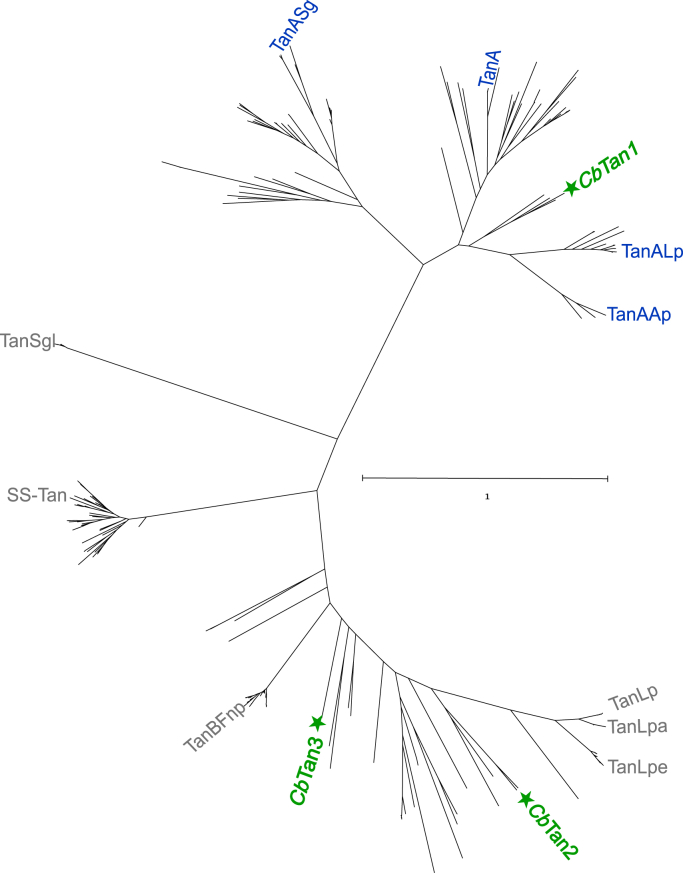
**Phylogenetic tree of characterized and putative bacterial tannase sequences.** Biochemically characterized tannases are named, and type A tannases are shown in *blue*, type B tannases in *gray*, and the tannases investigated in this study in *green* with a *star symbol*. The characterized tannases are: TanLp (*Lactiplantibacillus plantarum*), TanLpa (*Lactiplantibacillus paraplantarum*), TanLpe (*Lactiplantibacillus pentosus*), TanBFnp (*Fusobacterium nucleatum*), SS-Tan (*Streptomyces sviceus*), TanSgl (*Streptococcus gallolyticus*), TanASg (*Streptococcus gallolyticus*), TanA (*Staphylococcus lugdunensis*), TanALp (*Lactiplantibacillus plantarum*), TanAp (*Atopobium parvulum*). The IMG/M database accession numbers and locus tags can be found in Supplemental information 2 (https://img.jgi.doe.gov/). Total number of sequences: 260. The scale bar reflects numbers of substitutions per site.

*C. butyricum* encodes three putative tannases that were named *Cb*Tan1-3 (locus tags K670DRAFT_04079, K670DRAFT_03878, K670DRAFT_04016, respectively). The enzymes have as low as 31% sequence identity among each other, determined using the protein–protein BLAST algorithm (Table S1), but the reason for this multiplicity is currently unknown. *Cb*Tan1 and *Cb*Tan3 were predicted to have signal peptides, indicating them being secreted and active outside the cell, while *Cb*Tan2 might be an intracellular enzyme. Inspection of the genomic neighborhood surrounding each gene showed that *Cb*Tan1 is in vicinity of a putative gallate decarboxylase (locus tag K670DRAFT_04082), while no genes similarly predicted to be related to tannin metabolism were found in the vicinity *Cb*Tan2 or *Cb*Tan3. To investigate the similarity of the *C. butyricum* tannases to studied enzymes, a phylogenetic tree was constructed. Previously characterized bacterial tannases (Table S1) were BLAST searched against all genomes in the Integrated Microbial Genomes & Microbiomes (IMG/M) database, and the top 50 hits from each search were selected. Duplicate, partial, and signal peptide sequences were removed resulting in a total of 260 unique sequences, which were used to construct the phylogenetic tree (simplified in Fig. 2; full tree in Supplemental information file 2). The characterized fungal tannases and the enzyme Tan410 (discovered in a soil metagenome) were found to not have any sequence similarity to the bacterial enzymes, indicating that they form a separate tannase family and were thus excluded from the phylogenetic analysis (Table S1) (18). Within the tree, the lowest sequence identity between biochemically characterized tannases is as low as 24% and the highest 88% (Table S1). The three *C. butyricum* tannases were found to be located in different parts of the tree, reflecting their sequence diversity (Fig. 2). Furthermore, certain clades representing highly similar enzymes can be observed in the tree, which suggests correspondingly similar functions, though none of the *C. butyricum* enzymes fell into these distinct outgroups. The closest characterized homolog for *Cb*Tan1 is the TanALp enzyme from *L. plantarum*, (50% seq. id.), which has been shown to be inactive on long-chain alkyl galloyl esters but active on other tannase substrates (28). *Cb*Tan2 was most similar to the *Lactiplantibacillus* enzymes TanLpe, TanLpa, TanLp, (45–47% seq. id.) which have been found to be active on catechin galloyl esters, and *Cb*Tan3 was found in the middle of the tree close to both TanBFnp from *F. nucleatum* (52% seq. id.) and SS-Tan from *Streptomyces sviceus* (36% seq. id.), which have been found to be active on digallic and tannic acid. The varied level of characterization of these similar tannases precludes a functional prediction of the *C. butyricum* tannases, and to gain further insight into their properties, we chose to biochemically characterize them.

### Biochemical characterization

The genes encoding the three *C. butyricum* tannases were cloned and expressed in *E. coli*, and the resulting proteins were purified using affinity chromatography. *Cb*Tan1 (∼65 kDa) and *Cb*Tan2 (∼55 kDa) were purified to electrophoretic homogeneity (Fig. S1), though despite multiple efforts to purify *Cb*Tan3, the intact protein (55 kDa) could not be separated from a contaminating 30 kDa protein (Fig. S2*A*). The contaminating protein band was determined to have 20% intensity compared with the main protein band (using Image Lab v.6.0.1). To investigate if the 30 kDa protein exhibited tannase activity, the sample was incubated at 4 °C and samples taken up to 4 weeks, to evaluate if protein degradation correlated with enzyme activity. SDS-PAGE analysis of the samples showed a decreased intensity of the main band and apparent degradation into ∼44 and ∼30 kDa proteins, as well as a corresponding decrease in the activity of the enzyme over time (Fig. S2*B*). The analysis indicates that the main band of interest is responsible for the tannase activity and that the 30 kDa contaminant protein does not exhibit any prominent tannase activity. Taking the contamination into consideration, the kinetic constants of *Cb*Tan3 are thus reported as observed/apparent values (see below).

**Figure 3:**
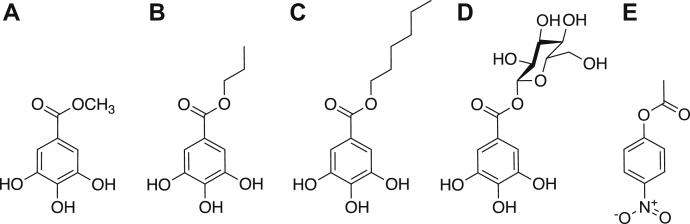
**Model substrates used to determine kinetic parameters.***A*) methyl gallate (MG), *B*) propyl gallate (PG), *C*) hexyl gallate (HG), *D*) β-Glucogallin (GG), all for determining tannase activity; and *E*) *para*-nitrophenyl acetate (*p*NP-Ac), used to assay acetyl esterase activity.

The enzymes were assayed on various tannase substrates (methyl gallate—MG, propyl gallate—PG, hexyl gallate—HG, and β-Glucogallin—GG; Fig. 3), using a modified rhodanine assay, which is based on the specific reaction of free gallic acid with rhodanine. Acetyl esterase activity was assayed on the acetyl esterase substrate *para*-nitrophenyl acetate (*p*NP-Ac) (29). The pH dependencies of the enzymes were assayed at a pH range between 3 and 10, using 1 mM MG, and the optimal pH for *Cb*Tan1 and *Cb*Tan2 was found to be 6 to 7 and for *Cb*Tan3 pH 7 (Fig. S3). These values are consistent with those reported for other bacterial tannases isolated from the gut and soil (19, 20, 24, 28). Kinetic parameters were determined at each respective pH optimum for all substrates where possible (Table 1).

**Table 1:** Kinetic parameters of *C. butyricum* tannases on model substrates

Enzyme	Substrate	*K*_*m*_ (mM)	*k*_*cat*_ (s^-1^)	*k*_*cat*_*/K*_*m*_ (s^-1^ mM^-1^)
*Cb*Tan1	MG	10.8 ± 1.18	26.9 ± 0.90	2.40 ± 0.33
	PG	12.0 ± 1.60	32.0 ± 1.00	2.60 ± 0.43
	GG	3.50 ± 0.70	213 ± 11.4	72.8 ± 22.2
	HG	Cannot be saturated up to 10 mM		3.60 ± 0.22
	*p*NP-Ac	Cannot be saturated up to 10 mM		0.07 ± 0.004
*Cb*Tan2	MG	23.4 ± 2.68	187 ± 7.43	7.99 ± 1.13
	PG	22.2 ± 2.26	136 ± 4.27	6.17 ± 0.76
	GG	23.0 ± 3.70	209 ± 13.4	10.2 ± 1.80
	HG	Cannot be saturated up to 10 mM		6.40 ± 0.22
	*p*NP-Ac	Cannot be saturated up to 10 mM		0.03 ± 0.002
*Cb*Tan3^a^	MG	24.3 ± 3.52	45.9 ± 2.2	1.64 ± 0.09
	PG	7.20 ± 0.90	15.9 ± 0.64	2.24 ± 0.30
	GG	8.99 ± 1.70	459 ± 29.0	52.9 ± 12.5
	HG	Cannot be saturated up to 10 mM		1.28 ± 0.004
	*p*NP-Ac	No detectable activity at 10 mM		

Tannase activity was measured using the rhodanine assay, and acetyl esterase activity using *p*NP-Ac. The enzymes showed no activity on methyl ferulate (data not shown). The standard deviation was determined from triplicate experiments and calculated using OriginPro software v. 9.6.0.172.

a As *Cb*Tan3 was not fully pure, the values represent *k*_*cat*_^obs^ and *K*_*m*_^obs^.

All enzymes were active on the tannase substrates and showed minimal or no activity on *p*NP-Ac (Table 1). *Cb*Tan1 had a threefold lower *K*_*m*_ on the GG substrate than MG and PG, indicating a preference for glucose rather than alkyl groups ester-linked to gallate, with a 20-fold higher *k*_*cat*_*/K*_*m*_ for GG than the other tested substrates. *Cb*Tan2 had similar *K*_*m*_ and *k*_*cat*_*/K*_*m*_ values on all tested substrates, indicating that this enzyme can accommodate a range of moieties linked to gallic acid. *Cb*Tan3 had a similar *K*_*m*_ on both PG and GG, which could indicate a preference toward “medium” sized substitutions on gallic acid. The *k*_*cat*_ for GG was however close to 30-fold higher than for PG, resulting in a 24-fold higher *k*_*cat*_/*K*_*m*_ value for GG, which demonstrates that both *Cb*Tan1 and *Cb*Tan3 have a clear preference toward glucose-containing substrates. Overall, both *Cb*Tan1 and *Cb*Tan3 had similar catalytic efficiencies on GG, though it must be considered that *Cb*Tan3 was not fully pure, meaning that its catalytic efficiently has been underestimated in this study. Fresh *Cb*Tan3 enzyme was used for each experiment to ensure minimal degradation, as described previously (Fig. S2). For the MG and PG substrates, the catalytic efficiency of all *Cb*Tan enzymes was within the same order of magnitude (Table 1).

There are very few other reports of tannase substrate preferences and kinetics. The three tannases (TanLp, TanLpa, TanLpe) from the three *Lactiplantibacillus* species *(L. plantarum, Lactiplantibacillus paraplantarum, and Lactiplantibacillus pentosus*) had *K*_*m*_ values ranging between 0.37 to 0.87 mM on MG (12). These enzymes showed a tenfold lower *K*_*m*_ on catechin derivatives, but no difference in the catalytic efficiency on catechin and methyl derivatives. In our study, the differences in *K*_*m*_ and *k*_*cat*_/*K*_*m*_ between different substrates were large for *Cb*Tan1 and *Cb*Tan3 but not for *Cb*Tan2. However, the *K*_*m*_ values on MG for the *C. butyricum* tannases were around tenfold higher compared with the *Lactobacilli* enzymes, which could point to different biological roles of the enzymes between these species. To our knowledge, the only other reported enzyme tested for digallate activity is SS-Tan (*S. sviceus*) and TanLp (*L. plantarum*) (13). For SS-Tan, significant differences in substrate preference were reported, with a tenfold higher *k*_*cat*_/*K*_*m*_ on digallate substrates than MG (13).

### Biochemical degradation of water-soluble oak bark extractives

**Figure 4:**
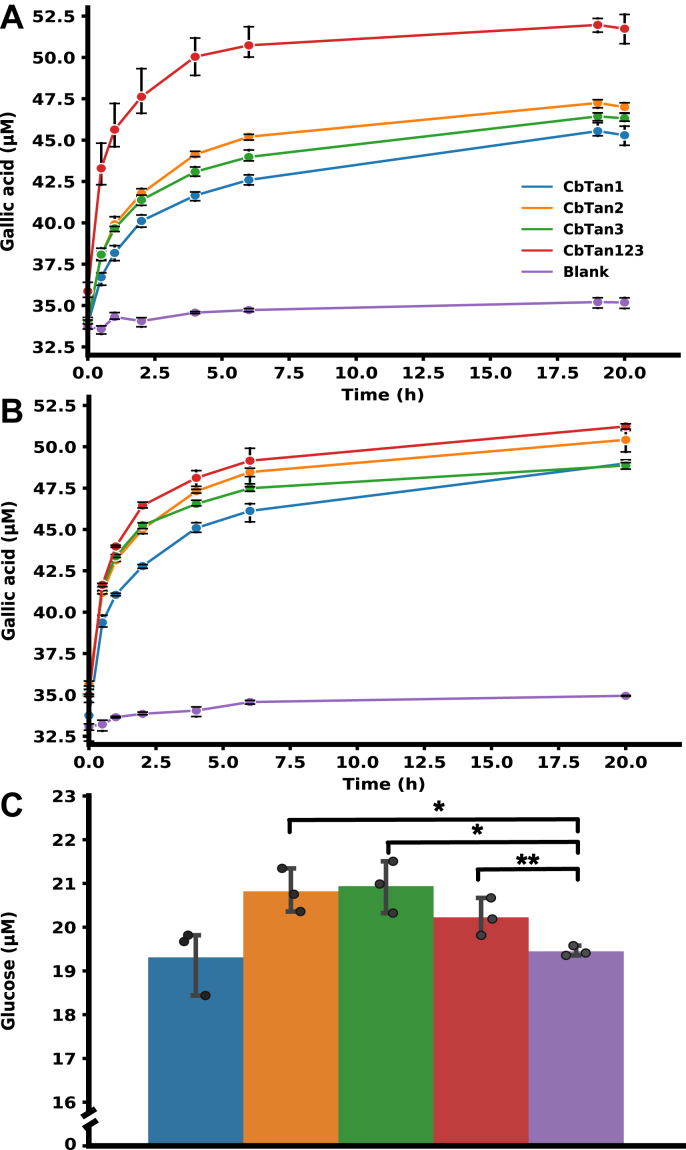
**Effect of tannase treatment on tannin containing water-extracted oak bark extract.***A*) Released gallic acid in the enzymatically treated oak bark extract over time compared to a sample containing no enzyme (blank), in reactions containing 0.04 nM of individual enzymes and 0.12 nM total enzyme for the reaction containing all enzymes. *B*) Released gallic acid in the enzymatically treated oak bark extract over time compared to a sample containing no enzyme (blank), in reactions containing 0.12 nM enzyme for both individual enzymes and when combined. *C*) Released glucose from the 20 h reactions, using the same color coding as in *A*. The stars indicated above the graph represent *p*-values of ∗≤0.01 or ∗∗≤0.05 as determined by a student *t-*test in Python. Reactions with *Cb*Tan2, *Cb*Tan3, and *Cb*Tan1-3 exhibited a statistically significant increase of released glucose compared to the blank sample (no added enzyme). There was no increase in glucose released from *Cb*Tan1 compared to the blank. The individual data points are shown, and also shown as means with standard deviations, from experiments on the same batch of oak bark extract.

The water-soluble extractives of oak bark are known to contain high concentrations of hydrolysable tannins, where a glucose unit is ester linked to several gallic acid or ellagic acid moieties. To explore whether the *C. butyricum* tannases were able to degrade these more complex structures, mild hot water extraction was performed on oak bark and the resulting extractives used as a substrate. As the bacterium encodes three tannases, our hypothesis was that they would act differently on this more complex substrate similarly to the differences observed on the model substrates. We tested the hypothesis by adding the enzymes separately at 0.04 nM to the extract and monitoring gallic acid release using HPLC-PDA. *Cb*Tan1 was found to be somewhat slower in the release of gallic acid compared with *Cb*Tan2 and 3 during the initial stages of the reaction, though after 20 h the total release was similar for all three enzymes. These reactions were compared to reactions containing the combined three enzymes at the same concentration as in the single-enzyme assays (total 0.12 nM tannase) and blank samples without enzyme. Reactions containing all three enzymes significantly increased the gallic acid released over time, but also indicated that the three enzymes did not act in apparent synergy on the substrate (Fig. 4*A*), as there was no large additive effect of gallic acid release in the sample with all enzymes. The combinations of *Cb*Tan1+2, *Cb*Tan1+3, and *Cb*Tan2+3 were also tested, with no additive effect observed (Fig. S4).

As the individual enzymes differed in their gallate release rates (Fig. 4*A*), a second hypothesis was that the enzymes favored hydrolysis of distinct parts of the bark extract. This was tested by repeating the single-enzyme assays (0.12 nM tannase) on a separate batch of water-extracted oak bark and comparing to a reaction containing all three enzymes but maintaining the total tannase concentration as in the single-enzyme reactions (0.04 nM each of *Cb*Tan1–3). Here, the addition of the three tannases simultaneously gave rise to a faster hydrolysis rate compared with the samples only containing one enzyme, indicating that they can complement each other and target different substrate moieties (Fig. 4*B*). *Cb*Tan1 was as before slower than the other enzymes and did not reach the same gallate release as the other enzymes until 20 h. In both sets of extractions, there was free gallate present in the blank sample, which is probably a by-product from the ball milling of the oak bark.

To monitor if the enzymes released any glucose from the water extract, as this is another component of gallotannins, glucose concentrations were measured in the 20 h samples using HPAEC-PAD. Interestingly, there was an increase in the glucose concentration for *Cb*Tan2, *Cb*Tan3, and the sample containing all enzymes (*Cb*Tan1-3) compared with the blank sample (Fig. 4*C*). The free glucose detected in the blank sample, similar to gallate, could be a by-product arising from the ball milling. Surprisingly, *Cb*Tan1 did not release any additional glucose compared with the blank sample and again behaved differently than *Cb*Tan2 and 3. Possibly, the bark extract tannin structures contain significant amounts of depsides, *e.g.*, as multiple ester-linked gallic acid moieties surrounding a central glucose unit, as in tannic acid. *Cb*Tan1 might then be limited to cleave gallic acid from the outer “shell” and unable to fully hydrolyze the structure to release glucose, in contrast to *Cb*Tan2 and 3, which also exhibited different gallic acid release rates as described above. *Cb*Tan1 and 2 were however active on the GG model substrate, and possibly gallic acid linked to other positions than the anomeric carbon or other moieties linked to the glucose may make these substrates inaccessible to the enzyme.

### Mass spectrometric analysis of water-soluble oak bark extractives

To gain further insight into the molecules present within the oak bark extract and enzyme-treated samples, the 20 h samples were analyzed by reversed-phase liquid chromatography coupled to a High-Resolution Mass Spectrometer (Waters Acquity I-class UPLC, Waters Vion IMS QTOF). Compounds were putatively identified using a library of tannins derived from literature (Table S3), and annotated if present in all of the triplicate experiments based on their molecular weight, CCS (collision cross section), and fragmentation pattern compared with literature or standards if available (Table S4). Gallic acid and ellagic acid were identified using standards and detected in all samples (Table 2). All three tannases were able to degrade the putatively identified hydrolysable tannins tri- and di-galloyl glucose as these compounds were identified in the blank but not in the enzyme-treated samples, indicating hydrolysis (Table 2). Surprisingly, methyl gallate and galloyl glucose were putatively identified in the blank sample, and reactions with *Cb*Tan1 and *Cb*Tan3, but not in the reaction containing *Cb*Tan2. This indicates that *Cb*Tan2 is able to hydrolyze both methyl gallate and galloyl glucose present in the oak bark extract, while the other tannase enzymes cannot.

**Table 2:** Putative identification of compounds in the oak bark extract

Putative identification	Blank	*Cb*Tan1	*Cb*Tan2	*Cb*Tan3
Methyl gallate	+	+	ND	+
Gallic acid^a^	+	+	+	+
Ellagic acid^a^	+	+	+	+
Galloyl glucose	+	+	ND	+
Digalloyl glucose	+	ND	ND	ND
Trigalloyl glucose	+	ND	ND	ND

Compounds are annotated as detected (+) or not detected (ND) in the triplicate experiments based on molecular mass, CCS, fragmentation, and standards if available (Table S4).

a Identified using standards.

### X-ray crystallographic structure of CbTan2 and molecular modeling of CbTan1 and 3

#### Overall structure of *Cb*Tan2

A multiple sequence alignment of the three *C. butyricum* tannases and those with solved structures (TanLp and FnTan) revealed several putative unique regions (inserts and/or deletions) in each protein (Fig. S5), though with the limited structural analysis of tannases to date, the possible roles of these regions are difficult to evaluate. In order to gain further insight into the determinants behind the different properties of the *C. butyricum* tannases, we pursued crystallization of all three enzymes. We were able to crystallize and solve the structure of *Cb*Tan2 extending to 2.22 Å resolution by molecular replacement using the structure of TanLp from *L. plantarum* as the search model (PDB code: 4J0C) (23), and data were deposited in the PDB with accession code 7Q6Y. Data collection and refinement statistics are shown in Table S6. Although crystals were obtained of *Cb*Tan2 when cocrystallized with gallate, the diffraction data obtained were of poor resolution (high resolution limit = 6 Å, data not shown), and we were unable to obtain better-diffracting crystals.

The apo structure of *Cb*Tan2 contained two protein molecules in the asymmetric unit (Fig. S6*A*). A single peak was observed using size-exclusion chromatography, and comparison to a calibration curve revealed that the protein was monomeric in solution (calculated MW ≈ 51 kDa *versus* expected MW ≈ 55 kDa), which suggests that the dimer observed in the structure is crystallographic and not of biological relevance. The overall structure adopted the α/β hydrolase fold (6 α-helices and 8 β-sheets) with multiple inserts that formed additional 12 helices and five β-sheets (Fig. S7), which is similar to the additional inserts observed in other crystallized bacterial tannases (22, 30). Chain A and B could be built from residues 1 to 490, with a single chain break in chain A and two in chain B. Residues from 116 to 135 (between β5 and α2, Fig. S7) could not be modeled in either chain as this region appears to be either poorly ordered or proteolytically cleaved. A disulfide bridge was observed between C320 and C332 in both chains; however, residues 321 to 331 (α7 to η4) could not be modeled in chain B due to lack of electron density in this region. These regions, which were not visible in the structure, appear to be unique to *Cb*Tan2, but are not proximal to the enzyme active site and thus are likely not directly involved in catalysis. The smaller than expected MW may be explained by these regions, which were possibly proteolytically cleaved.

**Figure 5:**
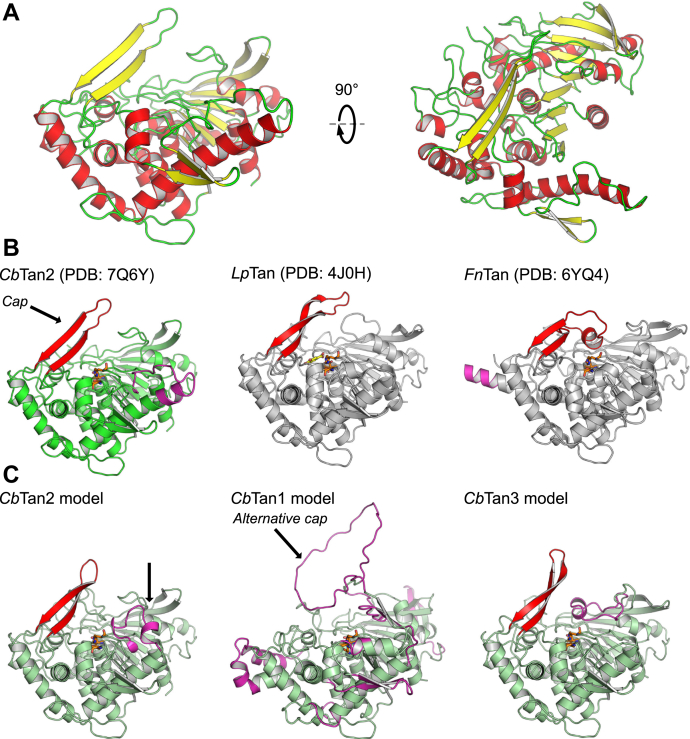
**Structure of *Cb*Tan2 and comparison to other bacterial tannase structures and models.***A*) The overall structure of a monomer of *Cb*Tan2, showing chain A in the crystallographic unit with secondary structural feature coloring (*yellow sheets*, *red helices*). *B*) Structure of *Cb*Tan2 (*carbons in green*) with the cap region indicated, and of the previously published TanLp and FnTan (*carbons in gray*). The catalytic triad is shown as sticks in *orange*, the cap region in *red*, and unique inserts in *magenta*. Gallate bound to TanLp is shown as a *yellow ball* and *stick* representation. *C*) Predicted structures of *Cb*Tan 1 to 3 using ColabFold (AlphaFold2-derived algorithm) (35), with the large insert of *Cb*Tan1 that might form an alternative cap region indicated. The disulfide bridge forming part of a helix in *Cb*Tan2 that was not predicted by ColabFold is indicated with a *black arrow*. Shown are the homology models (*left to right*) for *Cb*Tan2, *Cb*Tan1 and *Cb*Tan3 (carbons are *light green* for the models).

A hairpin-style cap region over the active site was identified in *Cb*Tan2, formed by residues 237 to 258, and comprised of two antiparallel β-strands connected by a turn in a hairpin (β8 & 9), similar to the cap identified in TanLp (Fig. 5, *A* and *B*) (23). We were able to model all cap residues in both chains (average B-factor over protein = 46.7, over cap region = 54.9, over loop residues 244–250 in cap = 65.0). Superposition of the two chains in the structure of *Cb*Tan2 showed slightly different positions of the cap (Cα RMSD of the two chains = 0.232). This appears to be a result of extensive crystal contacts between the cap domains of adjacent protein molecules (Fig. S6, *B* and *C*), and possibly these contacts may have influenced the conformation observed here. The cap domain has been previously reported in TanLp to be highly flexible based on the observation of weak density in the loop connecting the two β strands, but there were no major conformational changes between apo and substrate-bonded structures (23). It appears that the cap of *Cb*Tan2 adopts a similar shape and may also be flexible to help accommodate very large substrates, such as environmentally derived tannins that are more complex than the model substrates used here. The *Cb*Tan2 cap domain is markedly different compared with FnTan from *F. nucleatum*. Rather than containing two antiparallel β-sheets connected by a turn, as in *Cb*Tan2 and TanLp, the cap of FnTan contains an additional short α-helical region, which sits close to and strongly interacts with the surface of the substrate-binding cavity (Fig. 5*B*). Molecular dynamics simulations of FnTan suggested that the cap region initially opens and then closes over the substrate, forming interactions with the Tyr243 residue, unique to FnTan (22). Mutagenesis of several residues in the cap of FnTan either reduced (in the case of Tyr243) or did not significantly affect the catalytic efficiency of the enzyme, suggesting that these residues may contribute to stabilization of the cap region. It is possible that different evolutionary pathways of tannases have led to markedly different strategies for binding various substrates found among complex plant extractives.

#### Catalytic triad and gallate-binding site

**Figure 6:**
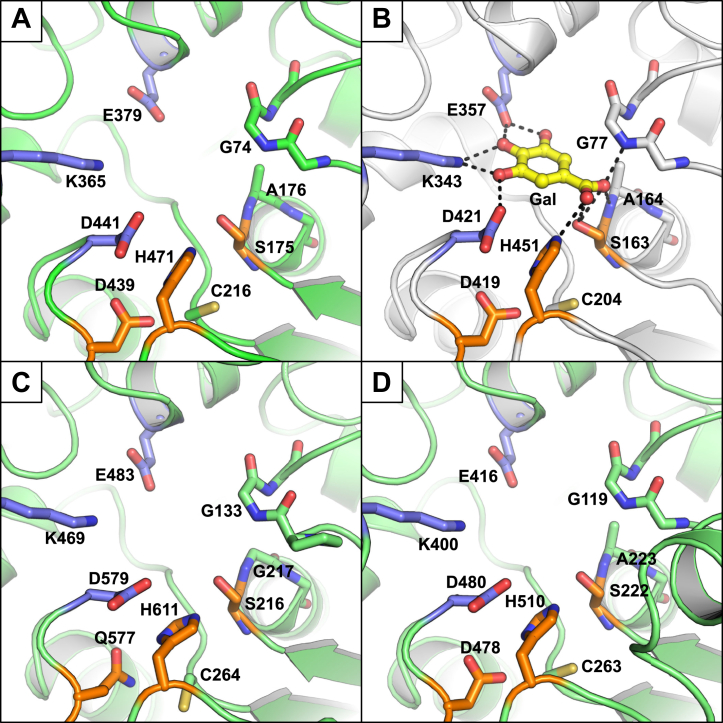
**Close-up views of tannase active sites.***A*) The newly solved *Cb*Tan2 active site (Chain A shown), with catalytic residues shown as sticks (*orange*) and predicted gallate binding residues (*purple*). *B*) The active site of gallate-bonded TanLp (PDB: 4J0H). Interactions to active site residues and oxyanion hole are shown as *dashed lines*. *C*) Homology model of *Cb*Tan1 active site, and *D*) of *Cb*Tan3 active site, both generated using ColabFold (35). In each figure, the cap domain, in front of the active site with respect to the view, has been hidden for clarity; additionally the section of peptide backbone, containing the likely oxyanion hole, is labelled and colored the same as the overall structure. Note the difference in residues in the catalytic triad and oxyanion hole of *Cb*Tan1.

The catalytic triad of *Cb*Tan2 could be identified as Ser175, Asp439, and His471 (Fig. 6*A*), which is consistent with the enzyme being a subtype B tannase, *i.e.*, possessing a catalytic Asp residue (16). The catalytic serine residue is found in the sequence motif G-X-S-X-G (Gly173-Thr174-Ser175-Ala176-Gly177), which is conserved across bacterial tannases (16). The catalytic histidine residue was oriented toward Ser175 and form a hydrogen bond (3.2 Å) as per the general mechanism of α/β-hydrolase enzymes (31). All of the catalytic triad residues were found in the same secondary structural motifs as in the canonical α/β hydrolase fold (Fig. S7). By superposition with the gallate-bonded structure of TanLp (PDB code: 4J0H), we propose the gallate-binding site of *Cb*Tan2 to be composed of Lys365, Glu379, and Asp441 (Fig. 6, *A* and *B*). Just below the catalytic histidine, Cys216 is present in a near identical location to Cys residues found in other bacterial tannases and could play a role in proton transfer to the catalytic triad as has been proposed previously (Fig. 6) (23). Catalysis by serine hydrolases relies on the so-called oxyanion hole for stabilization of the reaction intermediate (32) and is typically comprised of backbone nitrogen atoms, though exceptions with positively charged side chains have been identified (33, 34). In *Cb*Tan2, the oxyanion hole appears to be formed by the backbone nitrogen atoms of Gly74 and Ala176. The electron densities found in the active site of the two protomers in the crystallographic unit could be modeled as ordered water and 1,2-ethanediol molecules, which was a component of the crystallization buffer. The positions of all the residues comprising the catalytic triad and gallate-binding site were nearly identical between both protomers.

#### Homology models of *Cb*Tan1 and *Cb*Tan3

To gain possible deeper insight into the differences in substrate binding and activity between the *C. butyricum* tannases in the absence of experimentally determined crystal structures, we constructed homology models of *Cb*Tan1–3 using the free platform, ColabFold, which is based on a modified AlphaFold2 algorithm (35). The quality of each model is shown in Fig. S8. In the well-conserved regions and around the active site, and throughout the α/β hydrolase fold regions, there was a high predicted confidence in the model. The predicted model of *Cb*Tan2 was very similar to the solved crystal structure (Fig. 5, *B* and *C*, Cα RMSD = 0.524), as were the positions of key active site residues (Fig. 6, *A* and *B*), with one significant difference in the extended helix past residue 320 (Fig. S7, end of α7 to start of η4). Here, the disulfide bridge between Cys320 and Cys332 was not correctly predicted, most likely due to the fact that the starting models are based on currently published crystal structures (the crystal structure of *Cb*Tan2 was not provided to the ColabFold software). This appears to have resulted in a predicted kink in this helix compared with the structurally determined continued helix (Fig. 5*B*, *Cb*Tan2, and Fig. 5*C*, *Cb*Tan2 model). While the overall prediction quality of the *Cb*Tan1 and 3 models was somewhat lower than *Cb*Tan2 (Fig. S8), probably due to the insert regions with no known homology to available structures, we believe the predicted structures of *Cb*Tan1 and 3 to be valuable to discuss the activity differences observed between the enzymes.

Overall, the *Cb*Tan2 and 3 model structures were very similar to each other with only minor apparent differences, but while *Cb*Tan1 shares the same general fold, it also displays much larger inserted regions relative to the other enzymes (Figs. 5*B* and S5). The active sites of *Cb*Tan1 and 3 were similar to that of *Cb*Tan2, with the main differences being in the unique regions close to the surface of each enzyme (Fig. 5, *B* and *C*). A major difference in the *Cb*Tan1 homology model was that no apparent cap region over the active site was present, making the catalytic residues potentially surface-exposed. There was a large insert in proximity to the active site, which may possibly have a capping role (77 residues, from 360 to 437, Fig. 5*C*), though there were no predicted secondary structure elements in this region. Whether it is indeed a region of high disorder or flexibility cannot be stated without additional experimental support. It is also unclear without further investigation whether the different substrate preferences of *Cb*Tan1 can be attributed to the insert regions and lack of an obvious cap domain.

*Cb*Tan2 can be superimposed onto both the *L. plantarum* tannase (PDB code: 4J0H; Cα RMSD = 0.771) and the *F. nucleatum* tannase (PDB code: 6YQ4; Cα RMSD = 0.621). Apart from the nonconserved regions unique to each enzyme, all secondary structural features were found in comparable positions. Near-identical positions were also observed for the catalytic triad and gallate-binding residues (Fig. S9). The predicted catalytic triad of *Cb*Tan1 is Ser216, His611, and Gln577, and in *Cb*Tan3 the corresponding residues are Ser222, His510, Asp478. Similarly, the galloyl-binding sites appeared to be comprised of Asp579, Lys469, and Glu483 in *Cb*Tan1 and Asp480, Lys400, and Glu416 in *Cb*Tan3. These residues were all found in the expected secondary structural features as in the canonical α/β hydrolase fold (Fig. S7). It is currently unknown if the Asp residue found in the gallate-binding pocket may serve a dual role of catalysis and binding in subtype A tannases, where there is no acidic residue in the catalytic triad. In both cases, these probable galloyl-binding residues overlaid with highly similar positions to the equivalents in the structure of *Cb*Tan2 (Fig. 6, *C* and *D*). Our observations collectively support *Cb*Tan1 being a type A tannase (large ∼600 aa protein size, Gln substitution for catalytic Asp), while *Cb*Tan2 and *Cb*Tan3 being type B tannases (smaller ∼500 aa protein size, Asp in catalytic triad).

It has not been determined which, if any, residues in tannases form interactions with the alcohol-derived component of tannin substrates. Crystal structures with dodecyl gallate have been determined with TanLp, but electron density was only found for the first two carbons. Further, the ethyl moiety did not form any interactions with nearby aliphatic residues in the active site or the cap “underside,” facing the active site, despite the presence of a Phe243, on the underside of the cap and Leu79 in the active site (23). The space between the active site and cap is very large, and this may permit the entry of large and diverse tannin substrates. The *Cb*Tan2 structure, and the homology models of *Cb*Tan1 and 3, did not show any equivalent aliphatic residues either in the vicinity of the catalytic triad or on the underside of the cap. It is thus difficult to predict from the currently available information if the residues in the cap regions of the *Cb*Tan enzymes play any role in stabilizing an enzyme–substrate complex, and as such substrate-bound structures will be required to ascertain more details about substrate binding and preference in tannases. It is worth noting that the cap regions, or possibly equivalent insert in *Cb*Tan1, had lower prediction scores, which indicate flexibility (Fig. S9), but also that there were no observed major differences in the positions of the active site residues, which could explain the different substrate specificities between the *Cb*Tan enzymes.

## Discussion

While *C. butyricum* has been found to inhabit different environments, from guts to soils, it has not been experimentally tested as a metabolizer of tannin, and the reason for it encoding three tannases in different parts of its genome is unclear. Using SignalP, *Cb*Tan1 and *Cb*Tan3 were predicted to contain signal peptides, implying secretion, but possibly the enzymes could be anchored to the membrane surface or extracellular Gram-positive cell wall (30). *Cb*Tan2 lacks a predicted signal peptide and might thus be an intracellular enzyme. Tannins are large and structurally complex molecules, but if the bacterium produces both external and internal tannases, it may be possible that tannin degradation takes place both outside and inside the cell, though how and if tannins are transported through the cell wall and membrane is currently not known. The expected presence of an intracellular tannase could suggest that *C. butyricum* not only detoxifies tannins, but also metabolizes these compounds.

Currently, the mechanism of tannin transport is unknown. However, tannin degradation has been investigated in the bacterium *S. gallolyticus*, where both tannases and a gallate decarboxylase were expressed during growth on methyl gallate (24). It is likely that the three tannases and the gallate decarboxylases genes in *C. butyricum* are similarly expressed as a response to sensing of the presence of tannins. Possibly, the encoded enzymes all cooperate in tannin degradation, or alternatively, they are expressed in response to different types of tannins, and future transcriptomic studies may reveal such regulatory differences. *C. butyricum* is by no means the only bacterium with multiple tannases, and both *L. plantarum* and *S. gallolyticus* are known to encode two tannases each, which have been studied in separate studies (20, 24, 28), our study represents the first single study of three tannases from the same organism.

It has been suggested that tannases can be divided into two distinct subtypes, A or B, based on amino acid sequence and protein length, where subtype B members have been suggested to efficiently hydrolyze long alkyl chains (16). Although the two tannases *Cb*Tan2 and *Cb*Tan3 cluster into subtype B in the phylogenetic tree (Fig. 1) and can act on the HG substrate, both enzymes have similar catalytic efficiency on the methyl and the hexyl galloyl esters, not hydrolyzing the HG substrate more efficiently. *Cb*Tan2 did not show a clear preference toward any model substrates tested in this study, though it was most closely positioned to the three tannases from *Lactoplantibacilli* in the phylogenetic tree. These three tannases have been shown to have tenfold lower *K*_*m*_ values on catechin compared to methyl galloyl esters, while the catalytic efficiency on both substrates was the same (12, 13), and possibly also *Cb*Tan2 would show a low *K*_*m*_ on catechin galloyl esters.

Hydrolysable tannins are composed of a central glucose unit; therefore, it is of interest to investigate tannase preference toward a glucose galloyl ester substrate. *Cb*Tan1 and *Cb*Tan3 both demonstrated preference toward the glucose-containing GG substrate but are found at opposite ends in the phylogenetic tree (Fig. 1), with *Cb*Tan3 found close to both the tannase SS-Tan from *S. sviceus* and TanBFnp from *F. nucleatum* in the phylogenetic tree (Fig. 1). No substrate preference was reported for TanBFnp, but preference toward digallate compared with methyl galloyl esters has been reported for SS-Tan (13, 19). SS-Tan was not tested on glucose galloyl ester, but had a higher catalytic efficiency on tannic acid compared with digallic acid, where the former is a substrate containing a mixture of both depside and ester bonds (13). Possibly, both *Cb*Tan3 and SS-Tan are highly active on both depside and ester bonds. *Cb*Tan1 groups into subtype A, close to TanALp from *L. plantarum*, which has been reported to degrade esters derived from gallic and protocatechuic acids but not lauryl substituents (28). *Cb*Tan1 displayed no significant difference in catalytic efficiency between the alkyl galloyl esters, suggesting that preference for long-chain alkyl groups is not a key separator quality between subtype A and B tannases.

Correlation between placement in the phylogenetic tree and substrate preference is not entirely evident, and more studies focused on tannase substrate preference, and comparison of tannases from the same microorganism would aid to link amino acid sequence and substrate affinity. Based on our observations, it would appear that the suggested grouping of tannases into subtypes A and B does not have any actual functional bearing and is not a useful predictor of enzyme activity. As this subgrouping was proposed only using a few tannase sequences (16), a more thorough investigation of tannases from the entire phylogenetic tree would be highly informative. Tannases are highly diverse enzymes, at least in primary structure, and more functional and structural studies are necessary to better understand their properties and biological roles. Our model of *Cb*Tan1 highlights the need to better understand how large insert regions and presence or absence of a structured cap region influences the activity and biological role of these enzymes. It remains yet unclear if there are residues nearby to the catalytic triad or contained in the cap, which play a role in the substrate preference of each *Cb*Tan enzyme. It is also evident from this and other recent studies that the presence or absence of a predicted signal peptide is not a valid categorization of tannin subtypes.

Tannins are a group of molecules composed of numerous different molecular structures, which likely demand a range of enzymes for complete hydrolysis. Analysis of extractives from oak bark has shown that it contains a mixture of tannins (36, 37, 38), and the water-extracted oak bark used in this study is likely also comprised of a mixture of tannins. However, when treated with tannases, the extract was not degraded in any apparent synergistic manner between the three different *C. butyricum* enzymes. The results indicate that the enzymes prefer different parts of the extractives, as the rate of gallic acid release differed for all individual enzymes, and the sample containing all enzymes gave rise to the fastest gallic acid release. Only in the later stages did the reactions reach the same level of hydrolysis, likely as an effect of the preferred substrates being depleted and less preferred ones being targeted. Glucose was released in reactions containing *Cb*Tan2 and/or *Cb*Tan3, indicating that these tannases can cleave all ester bonds in gallotannins found in the extract, in contrast to *Cb*Tan1. On the extract, all three enzymes were able to hydrolyze the larger hydrolysable tannins putatively identified using high-resolution MS. In addition, *Cb*Tan2 was also able to hydrolyze the putatively identified methyl gallate and galloyl glucose, even if both *Cb*Tan1 and *Cb*Tan3 could degrade these model substrates. This lack of activity for *Cb*Tan1 and *Cb*Tan3 could be due to many different reasons, such as substrate inhibition, or positioning of the galloyl ester on the glucose, and it is interesting to speculate whether the large inserts predicted for the enzyme play a role in substrate access or positioning that limits the enzymes’ ability to reach full hydrolysis.

Taken together, the activity on both model substrates and oak bark extract show that the *C. butyricum* tannases *Cb*Tan1 and 3 act similarly on both model substrates and the oak bark extract, displaying a preference for glucose-containing substrates and similarly degrading putatively identified compounds from bark. In addition, the glucose release from *Cb*Tan3-treated oak bark extract indicates that *Cb*Tan3 can degrade additional substrates present in the oak extract, though identification of the exact nature of these was not possible in this study. *Cb*Tan2 was able to hydrolyze more of the oak bark tannin compounds compared with the other two tannases. Together, our data indicate that the *C. butyricum* tannases are functionally different and possibly have different biological roles. The prediction that *Cb*Tan1 and 3 are secreted while *Cb*Tan2 resides within the cell could also suggest that *Cb*Tan2 and 3 perform similar functions but in different cellular locations, while *Cb*Tan1 performs a different function in the extracellular milieu.

*C. butyricum* is used as a probiotic and has been shown to change the microbiome profile (39). Possibly this is to an extent tied to its presumed ability to remove and/or detoxify tannins in both gut and soil environments and thus, is able to support not only its own growth but also that of neighboring cells. Whether this is the case, in a similar vein to gut species enabling cross-feeding on complex carbohydrates or not remains to be elucidated (40), but it is however likely that the *C. butyricum* tannases facilitate its competitiveness in tannin-rich environments. Previous studies on microorganisms encoding multiple esterase enzymes of the same type, such as feruloyl and glucuronyl esterases, have shown that the enzymes typically display quite different substrate preferences (33, 41), and similarly, this first study of multiple tannases from the same microorganism follows that trend. Future studies on the multiplicity of tannases in different microorganisms will help further shed light on specific biological roles and possibly also lead to improved utilization of these enzymes in industrial settings.

## Conclusion

In this work, we have shown that the three putative tannases from *C. butyricum* are functional enzymes with different substrate preferences on model substrates. *Cb*Tan1 and *Cb*Tan3 preferred the glucose galloyl ester substrate, while *Cb*Tan2 showed no preference for the type of substitution on gallic-acid-derived substrates used in this study. Further studies may include additional model substrates such as digallic acid or catechin, to possibly further delineate substrate preferences. The crystal structure of *Cb*Tan2 was solved to 2.22 Å resolution and conformed to the expected α/β hydrolase fold, while also possessing a cap covering the active site as seen in the two previously solved bacterial tannase structures. Homology models of *Cb*Tan1 and 3 suggest that while the three enzymes have a shared core, they differ significantly in their overall structures due to various inserts, and future studies may reveal whether these also govern the different activity profiles of the three enzymes. No apparent synergy was observed when adding the enzymes together on water-soluble oak bark extractives. The individual enzymes however gave rise to different rates of hydrolysis, and the observation that *Cb*Tan2 and *Cb*Tan3 were able to release glucose from extractives in contrast to *Cb*Tan1 and that *Cb*Tan2 was able to degrade substrates, which the other enzymes could not hydrolyze, points toward each enzyme having a unique biological role in *C. butyricum*.

## Experimental procedures

### Phylogenetic tree

The phylogenetic tree was created by using Basic Local Alignment Search Tool (BLAST) with characterized tannase sequences against the Integrated Microbial Genomes & Microbiomes (IMG/M) database with an E-value of 1e^−5^, selecting the 50 top hits for each sequence. Duplicate sequences were removed and SignalP v 5.0 was used to determine which signal peptide sequences to remove (30). The sequences were aligned using MUSCLE (42) and the phylogenetic tree was generated with IQ-TREE using 1000 ultrafast bootstrap approximation, including automatic identification of best-fit substitution model (WAG+F+I+G4) (43). The generated phylogenetic tree was visualized using MEGA-X v. 10.1.8.

### Cloning, expression, and purification of tannases

The coding regions of the tannases were amplified by PCR (primers in Table S2) from the genomic DNA of *C. butyricum* DSM 10702. The vector pET-28a-TEVc was cut with the FastDigest restriction enzymes NdeI and XhoI (Thermo Fisher Scientific) and purified with GeneJet Gel Extraction Kit (Thermo Fisher Scientific) prior to incorporation with the tannase-encoding fragments using the In-Fusion HD Cloning Plus Kit (Takara Bio Inc) and transformation into chemically competent Stellar (Takara Bio Inc) cells. Plasmid DNA was purified with GeneJet Plasmid Miniprep Kit (Thermo Fisher Scientific) and sequenced to verify correct tannase incorporation. Plasmids were then transformed into chemically competent *E. coli* BL21(λDE3) for gene expression. The cells were precultured in 10 ml Lysogeny broth (LB) with 50 μg/ml neomycin for 24 h, 37 °C, 200 rpm, and then propagated in 1 L LB until they reached an OD_600_ of 0.4 to 0.6, when protein expression was induced with 0.5 mM isopropyl β-d-1-thiogalactopyranoside. The cells were incubated for 72 h at 17 °C and harvested by centrifugation. Cell pellets were resuspended in 20 mM tris(hydroxymethyl)aminomethane (TRIS), pH 8, 250 mM NaCl, with 5 μg/ml lysozyme and 10 μg/ml DNase and disrupted using sonication and resulting cell debris removed by centrifugation. The proteins were purified by immobilized metal affinity chromatography (IMAC) using a 5 ml HisTrap Excel column (Cytiva), using an ÄKTA FPLC system. The column was first washed with 5 column volumes (CV) of loading buffer with 50 mM TRIS, pH 8, 250 mM NaCl at a rate of 1 ml/min. The proteins were eluted using a 0 to 100% gradient of 250 mM imidazole in 50 mM TRIS, pH 8, 250 mM NaCl. Fractions of flow-through, wash, and elution were collected and evaluated for purity using pre-cast stain free sodium dodecyl sulfate–polyacrylamide gels for electrophoresis (SDS-PAGE) (Bio-Rad). A Nanodrop 2000 spectrophotometer (Thermo Fisher Scientific) was used to determine protein concentration using the predicted values for molecular weight and extinction coefficient (Expasy ProtParam server).

### Biochemical characterization

The activities of the tannases were determined using a modified rhodanine assay (29), enabling gallic acid detection *via* the formation of a complex with the rhodanine under alkaline conditions, with decreased volumes but keeping the solvent ratios. The tannase activity was determined in 200 μl reactions using 1 mM methyl gallate (MG) in 100 mM buffer and incubated at 5 min at 37 °C. Next, 130 μl methanolic rhodanine solution (0.667% w/v) was added and incubated for 5 min at 37 °C. Finally, 70 μl 0.5 M potassium hydroxide was added, and the reaction was incubated for 5 min at 37 °C. The absorbance was measured at 520 nm in a plate reader (BMG LABTECH). One unit of tannase activity was defined as the amount of enzyme required to release 1 μmol of gallic acid per minute under standard reaction conditions. The concentration of gallic acid released was determined using an external standard curve (1–27.5 μM).

The pH optima for the tannases were determined using the rhodanine assay, varying the 100 mM buffer used (sodium citrate pH 3.0–6.0, 2-(*N*-morpholino)ethanesulfonic acid (MES) pH 5.0 to 6.0, sodium phosphate pH 6.0 to 8.0, bis-(2-hydroxyethyl)amino-tris(hydroxymethyl)methane (BIS-TRIS) pH 6.0 to 7.0, TRIS pH 8.0 to 9.0, bicine pH 8.0 to 9.0, *N*-cyclohexyl-2-aminoethanesulfonic acid (CHES) pH 9.0 to 10.0, 4-(2-hydroxyethyl)-1-piperazineethanesulfonic acid (HEPES) pH 9.0 to 10.0) and incubated as described above, Fig. S3.

The assays for kinetic parameter determinations were conducted at each enzyme’s pH optimum. Kinetic parameters were obtained by varying the concentrations of the substrates MG (0.5–160 mM), propyl gallate (PG) (1–130 mM), Hexyl gallate (HG) (0.2–5 mM), β-Glucogallin (GG) (0.5–100 mM), all purchased from Carbosynth. The following molar amounts of enzyme were used for kinetic determination: *Cb*Tan1 (0.0081–1 nmol), *Cb*Tan2 (0.046–0.09 nmol), *Cb*Tan3 (0.003–0.09 nmol). Controls to ensure that the kinetics measurements were done under initial (linear) rates were done by sampling four time points at 0, 1, 3, 5 min for the highest and lowest substrate concentrations used in the reactions.

For the determination of acetyl esterase activity, *p*-nitrophenyl acetate (*p*NP-Ac; Sigma) was used in 200 μl reaction assays performed at 37 °C with 100 mM buffer and varying amounts of substrate (1.25–10 mM), from stock solutions in 96% ethanol. The formation of *p*-nitrophenol was monitored at 405 nm over 5 to 30 min. The obtained data were analyzed using Origin software using nonlinear regression and linear regression in reactions where substrate saturation could not be reached.

### Hydrolysis of oak bark

Oak bark was stripped from a branch in Blekinge (southern Sweden), frozen in liquid nitrogen, and ball-milled in a TissueLyser II (QIAGEN) at 30 Hz for 30 s. The ball-milled oak bark was then subjected to mild water extraction using a mass-per-volume ratio of oak bark to water of 25 mg/ml and incubation for 1 h at 37 °C. After extraction, the suspension was centrifuged to remove bark particles. The water-soluble extractives were collected by decanting and diluted fivefold, then incubated with the *C. butyricum* tannases in reactions containing 100 mM BIS-TRIS, pH 6, and compared with control reactions containing no enzyme. Reactions were stopped using a 10 kDa cutoff filter (Amicon Ultra) and the flow-through was analyzed by HPLC.

Gallic acid released from the water-soluble oak bark extract was monitored using a Luna (5 μm, 250 × 4.6 mm, Phenomenex) column. The gallic acid was separated using a water-acetonitrile (ACN) gradient system containing 0.1% formic acid with a flow of 1 ml/min, and the column temperature was set to 25 °C. The gradient steps consisted of 10% ACN for 2 min, followed by a linear increase to 40% ACN over 8 min, and 40% ACN held for 5 min. Thereafter, the column was equilibrated at 10% ACN for 4 min. The gallic acid concentration was determined at 280 nm against a standard curve using pure external standards, and peak analysis was performed using ChromNav v.2.

Glucose release from the oak bark extract was monitored using high-performance anion-exchange chromatography with pulsed amperometric detection (HPAEC-PAD) on an IC5000 system (Thermo Scientific). A 2 × 250 mm Dionex Carbopack PA1 column (Thermo Scientific) with a 2 × 50 mm guard column (Thermo Scientific) was used at a column temperature of 30 °C. The eluents were A – 100% water, B – 200 mM NaOH, and C – 200 mM NaOH and 170 mM NaAc. The samples were eluted using eluent A (0.26 ml/min) and detected using a postcolumn addition of 200 mM NaOH (0.13 ml/min). Thereafter, the column washed with eluent C and equilibrated with eluent A. The glucose concentration was determined against a standard curve using a pure glucose external standard (1–20 mg/L). Peak analysis was performed using Chromeleon software 7.2.10 (Thermo Scientific).

### Identification of water-soluble oak bark extractives

The 20 h enzyme treated samples and the blank sample were analyzed by reversed-phase liquid chromatography and High-Resolution Mass Spectrometry to elucidate the tannin structures present in the oak bark extract. The samples were run on a C18 column (Waters Acquity UPLC BEH C18) using a ACN gradient with addition of 0.1% formic acid on a Waters ACQUITY UHPLC I-Class system coupled to a Waters VION IMS QTof, (ion mobility hybrid Quadrupole Time-of-Flight). For the mass spectrometer, an electrospray ionization interface was used operating in High Definition MS^E^ and negative (ESI-) mode. Equipment control and data acquisition and processing were performed using Waters UNIFI software (UNIFI, Version: 1.9.4–053 Waters). LC and MS settings are reported in Table S5.

The compounds were putatively identified with the aid of UNIFI software (UNIFI, Version: 1.9.4–053 Waters) using a library with tannin molecules identified from literature (Table S3). The molecular masses and structure were matched in the software by calculating accurate mass on target structure and fragment structures. The identity of compounds could be confirmed by comparing fragmentation, retention, and CCS with a commercially available standard, if no standard was available, the putative identity of molecules was assigned by comparing the fragmentation and CCS with literature or the AllCCS database. The criteria used to match a target to a component in the data were: mass error <5 ppm, CCS <2% or CCS <5% when predicted CCS values were used.

### Protein crystallography

Prior to crystallization, *Cb*Tan2 was further purified using size-exclusion chromatography using a HiLoad 16/600 Superdex 200 pg size-exclusion column (Cytiva), operated by an ÄKTA Explorer FPLC using 25 mM Tris pH 8.0 as buffer. Comparison with a calibration curve of protein standards confirmed that the enzyme was monomeric. The enzyme was concentrated using an Amicon 10 kDa cutoff centrifugal filter unit to 50 mg/ml (Nanodrop) and subjected to crystallization screening using a Mosquito robot to prepare sitting-drop vapor diffusion trays at 20 °C. Drops contained 0.3 μl protein + 0.3 μl reservoir condition, with a 40 μl reservoir volume, using the JCSG+, Morpheus, and PACT Premier screening kits (Molecular Dimensions). Crystals were obtained in 3 weeks from condition A11 from PACT Premier (0.2 M calcium chloride dihydrate, 0.1 M sodium acetate, pH 5.0, 20% PEG 6000, 25 mg/ml protein). Crystals were cryoprotected by soaking in a solution of 2:1:1 ratio of reservoir solution:50% w/v PEG 6000:100% w/v glycerol, for 5 min, then flash-cooled in liquid nitrogen. Diffraction data were obtained on 18th September 2021 on beamline ID23-2 at the European Synchrotron Radiation Facility at 100 K using a Dectris PILATUS3 X 2M detector. The dataset was collected at 0.1° increments over 360° and was processed using XDS (44).

### Structure determination of CbTan2 and modeling of CbTan1-3

The structure of *Cb*Tan2 was solved by molecular replacement with Phaser in the Phenix suite of programs (45), using the structure of *L. plantarum* tannase as the search model (PDB code: 4J0C) (23). The solvent content indicated two molecules in the asymmetric unit. The protein structure was manually rebuilt after phasing using Coot (46), and refinement performed using Phenix Refine (45), with several rounds of refinement and rebuilding. R_free_ was monitored using 5% of the diffraction data selected at random prior to refinement. Detailed data collection and refinement statistics are outlined in Table S6. The coordinates for the structure were validated and deposited in the PDB with accession code 7Q6Y. Structure prediction models of *Cb*Tan1–3 were produced using AlphaFold2 as modified and hosted by ColabFold, available online at https://github.com/sokrypton/ColabFold (35, 47, 48, 49, 50).

## Data availability

The structural data have been deposited to the Protein Data Bank with accession code 7Q6Y. The ColabFold models are available upon request. The other data are available in the manuscript.

## Supporting information

This article contains supporting information (42, 51, 52, 53, 54, 55). Supplemental information file 1 contains Tables S1–S6 and Figs. S1–S9. Supplemental information file 2 contains the full phylogenetic tree.

## Conflict of interest

The authors declare that there is no conflict of interest with the contents of this article.

## Supporting information

Supplemental Tables S1−S6 and Figures S1–S9

Supplementary Information File 2

NewCB2_refine_35.cif

NewCB2_refine_35.mtz

NewCB2_refine_35.pdb
