# Supplementary material for: Structural diversity and substrate preferences of three tannase enzymes encoded by the anaerobic bacterium Clostridium butyricum
Source: J Biol Chem. 2022 Feb 21;298(4):101758. doi: 10.1016/j.jbc.2022.101758 (PMC8958541; doi:10.1016/j.jbc.2022.101758)
Supplement: Supplemental Tables S1−S6 and Figures S1–S9 [file mmc1.pdf]

Supplemental information

Clostridium butyricum encodes three functionally and structurally diverse tannase enzymes active on water-soluble oak bark extractives

Authors: Amanda Sörensen Ristinmaa[1], Tom Coleman[1], Leona Carla Cesar[1], Annika Langborg Weinmann[2], Scott Mazurkewich[1,3], Gisela Brändén[4], Merima Hasani[3,5], and Johan Larsbrink[1,3,*]

Affiliations: [1]Division of Industrial Biotechnology, Department of Biology and Biological Engineering, Chalmers University of Technology, SE-412 96 Gothenburg, Sweden

[2]Early Chemical Development, Pharmaceutical Sciences, AstraZeneca, Gothenburg, Sweden

[3]Wallenberg Wood Science Center, Chalmers University of Technology, SE-412 96 Gothenburg, Sweden

[4]Department of Chemistry and Molecular Biology, University of Gothenburg, SE-405 30 Gothenburg, Sweden

[5]Division of Forest Products and Chemical Engineering, Department of Chemistry and Chemical Engineering, Chalmers University of Technology, SE-412 96 Gothenburg, Sweden

[*]Corresponding author: johan.larsbrink@chalmers.se

Table S1. Percent sequence identity and percent query coverage (in brackets) between all characterized tannase enzymes, calculated using BLAST (https://blast.ncbi.nlm.nih.gov/Blast.cgi). Type A tannases are shown in blue and type B tannases in grey. No sequence identity was obtained between the fungal enzymes (marked in orange), the metagenomics-derived Tan410 and the bacterial enzymes, using an E-value threshold of 0.05. This lack of identity is represented by blank fields. Genbank accession numbers are shown next to each enzyme name in the vertical column.

	**TanAAp**	**TanALp**	**TanASl**	**TanASg**	**TanBFnp**	**TanLpa**	**TanLpe**	**TanLp**	**TanSg1**	**SS-Tan**	**CbTan1**	**CbTan2**	**CbTan3**	**AoTan**	**AnFaeJ**	**AnTan**	**AsTan**	**ATAN1**	**Tan410**
**TanAAp (ACV51449.1)**	100.00 (100)	38.38 (96)	40.64 (99)	36.95 (92)	26.34 (88)	26.88 (90)	26.15 (89)	24.82 (92)	27.32 (97)	34.30 (60)	40.10 (95)	25.55 (90)	29.70 (76)						
**TanALp (WP_003640628.1)**	38.38 (98)	100.00 (100)	53.22 (93)	47.37 (93)	31.33 (91)	31.54 (86)	30.27 (96)	31.21 (88)	32.21 (95)	37.61 (71)	50.17 (96)	31.04 (86)	30.16 (83)						
**TanASl (CBI13425.1)**	40.00 (99)	51.29 (93)	100.00 (100)	46.17 (92)	34.30 (28)	26.35 (94)	33.76 (88)	26.62 (94)	28.04 (98)	28.14 (69)	48.74 (96)	29.95 (93)	27.46 (90)						
**TanASg (CBI13425.1)**	36.25 (94)	47.37 (93)	46.34 (93)	100.00 (100)	32.82 (86)	32.97 (81)	33.11 (89)	32.18 (87)	33.56 (95)	33.65 (90)	47.77 (95)	34.88 (81)	31.85 (85)						
**TanBFnp (ALQ42581.1)**	26.34 (88)	31.33 (90)	30.52 (90)	33.13 (74)	100.00 (100)	44.49 (99)	42.77 (99)	45.10 (99)	36.18 (95)	35.57 (95)	32.59 (76)	45.84 (99)	52.95 (94)						
**TanLpa (BAN10246.1)**	26.88 (89)	31.54 (72)	27.23 (91)	28.88 (86)	44.49 (97)	100.00 (100)	73.19 (100)	88.06 (100)	35.45 (96)	34.76 (95)	30.84 (79)	46.75 (99)	42.47 (95)						
**TanLpe (BAN10247.1)**	26.15 (88)	30.27 (84)	27.80 (90)	33.33 (76)	42.77 (97)	73.19 (100)	100.00 (100)	71.70 (100)	33.33 (96)	36.10 (95)	28.81 (88)	44.85 (99)	42.54 (95)						
**TanLp (AB379685.1)**	24.82 (91)	31.21 (75)	27.45 (91)	29.16 (89)	45.10 (97)	88.06 (100)	71.70 (100)	100.0 0 (100)	34.50 (96)	34.94 (95)	30.65 (78)	47.56 (99)	42.27 (95)						
**TanSg1 (WP_01296222.1)**	27.32 (92)	32.21 (80)	28.04 (92)	33.56 (80)	36.18 (91)	35.45 (93)	33.33 (94)	34.50 (94)	100.00 (100)	36.48 (68)	37.46 (83)	33.40 (94)	34.14 (89)						
**SS-Tan (AB379685.1)**	34.30 (62)	29.53 (76)	27.92 (76)	38.69 (65)	39.10 (77)	34.62 (94)	35.54 (92)	35.02 (94)	36.48 (78)	100.00 (100)	43.20 (62)	30.10 (95)	36.40 (91)						
**CbTan1 (WP_035765380.1)**	39.93 (95)	50.00 (94)	48.91 (95)	48.28 (93)	32.59 (88)	30.95 (96)	28.81 (96)	30.65 (95)	37.46 (98)	32.62 (91)	100.00 (100)	31.13 (85)	33.23 (86)						
**CbTan2 (WP_002581587.1)**	25.55 (88)	31.04 (74)	30.12 (90)	34.88 (68)	45.84 (97)	46.75 (98)	44.85 (98)	47.56 (98)	33.40 (96)	30.24 (95)	31.13 (73)	100.00 (100)	44.14 (94)						
**CbTan3 (WP_035765275.1)**	29.70 (70)	30.16 (56)	28.60 (90)	27.58 (95)	52.95 (96)	42.27 (99)	42.33 (99)	42.06 (99)	34.14 (95)	35.58 (98)	33.23 (59)	43.95 (99)	100.00 (100)						
**AoTan (BAE65552.1)**														100.00 (100)	27.61 (77)	26.94 (93)	24.60 (90)	49.83 (95)	25.40 (80)
**AnFaeJ (XP_001397058.1)**														27.61 (68)	100.00 (100)	27.46 (86)	46.79 (100)	24.60 (77)	28.08 (93)
**AnTan (An11g01220)**														27.46 (90)	27.46 (85)	100.00 (100)	26.54 (93)	28.40 (86)	31.34 (84)
**AsTan_41271 (OJJ62580.1)**														23.45 (70)	46.79 (79)	26.54 (73)	100.00 (100)	25.12 (65)	31.50 (72)
**ATAN1 (CAT07374.1)**														49.83 (100)	24.60 (85)	28.40 (92)	25.12 (90)	100.00 (100)	24.46 (76)
**Tan410 (ADU32682.1)**														26.24 (85)	28.08 (99)	30.92 (91)	31.50 (99)	24.46 (77)	100.00 (100)

Table S2. Primers used for amplification of *C. butyricum* tannase genes.

Gene	Primer	DNA sequence (5'-3')
*Cb*Tan1	CbTan1f	CTTCCAGGGCCATAGTTGTGGAAATACCTCTAAATC
	CbTan2r	TGGTGGTGCTCGAGTCTACTTCAAGCATTCATCAACC
*Cb*Tan2	CbTan1f	CTTCCAGGGCCATAGTATGCTTATTTTTGATGATAAAAATTATAAAGTA
	CbTan2r	TGGTGGTGCTCGAGTCTATTACTTAGTATATATTTCATCAATCCATGC
*Cb*Tan3	CbTan1f	CTTCCAGGGCCATAGTAGTAACAGTAAAAATTTAACTAGTG
	CbTan2r	TGGTGGTGCTCGAGTCTATTATTCTGAAATGCTGTCCATCC

Table S3. Library of tannins previously found in oak bark or tannin rich biomass.

1-digalloyl glucose	Pedunculagin
1,2,3,4,6-penta-*O*-galloyl-α-D-glucopyranose	*p*-coumaric acid
Acutissimin A	*p*-hydroxybenzoic acid
Acutissimin B	*p*-hydroxybenzoic aldehyde
Caffeic acid	ProcyanidinB2
Castalagin	Procyanidin B2 3'-*O*-gallate
Catechin	Protocatechuic acid
Catechin gallate	Protocatechuic aldehyde
Chlorogenic acid	Punicalagin
Coniferyl aldehyde	Quercetin
Corilagin	Quinic acid
Coumarin	Roburin A
Digallic acid	Roburin B
Ellagic acid	Roburin C
Epicatechin	Roburin E
Epigallocatechin Gallate	Scopoletin
Eucaglobulin	Shikimic acid
Eugenol	Sinapic acid
Ferulic acid	Sinapic aldehyde
Fructose	Sucrose
Gallic acid	Syringic acid
Gentisic acid	Tannic Acid
Glucogallin	Trans-resveratrol
Glucose	Trigalloyl glucose
Grandinin	Valoneic acid dilactone
Hexahydroxydiphenic acid	Vanillic acid
Methyl 4,6-di-*O*-galloyl-β-D-glucopyranoside	Vanillin
Methyl gallate	Vescalagin
Myricetin	

Table S4. Identified compounds in the enzyme treated samples and the untreated sample (blank). Compounds are only reported if found in all triplicate experiments in either enzyme treated or untreated samples with a retention time (RT) of ΔRT of <0.07 min. The criteria used to match a target to a component in the data were: mass error <5 ppm, CCS <2% or CCS <5% when predicted CCS values were used. Fragment ions shown in other studies are underlined.

RT	Mass (m/z)	Main fragment ions (m/z)	CCS_{meas} (CCS_{ref})	Tentative identification	Formula	Exact mass	Mass error (ppm)	Literature reference CCS_{ref} + fragment ions
0.93	169.0146	125.0249	126 (125)	Gallic acid*	$C_7H_6O_5$	170.0215	1.9	This study
0.95	331.067	169.0146, 211.0249, 315.0721, 125.0253	175 (175)	Galloyl glucose[1]	$C_{13}H_{16}O_{10}$	332.0743	-1.2	AllCCS + (1)
2.28	483.0777	271.0458, 211.0255, 313.0576, 331.0657	199 (199.3)	Digalloyl glucose[1]	$C_{20}H_{20}O_{14}$	484.0853	-0.6	AllCCS + (1) + (2)
2.5	635.0888	483.076, 437.071, 295.047,	227 (227.9)	Trigalloyl glucose	$C_{27}H_{24}O_{18}$	636.0963	-0.3	(1,3)
2.57	183.0304	124.0170, 168.0064, 125.0242,	134 (136.2)	Methyl gallate[1]	$C_8H_8O_5$	184.0372	3.0	AllCCS + (4)
4.21	300.9993	169.0147, 257.0095, 283.9968	156 (155)	Ellagic acid*	$C_{14}H_6O_8$	302.0063	1.1	This study

*Identified using standards

[1] predicted CCS

Table S5. Instrument settings for the LC-MS analysis.

Setting/equipment	
Column	Waters Acquity UPLC BEH C18 (1.7 µm, 100 × 2.1 mm, Waters)
Mobile phases	A: Water/Formic acid 100/0.1 B: ACN/Water/Formic acid, 95/5/0.1
Gradient	5-95% B in 9.5 min, 40-99B% in 0.2min, 99%B for 0.7min, 5%B for 1.1 min
Sample volume	7 µL
Column temperature	45 °C
Flow rate	0.4 mL/min
MS Make up pump	0.375 ml/min
Ionization source	ESI-
Scanning range	50-2000 m/z
Scanning time	0.2
Capillary voltage	2.5 kV
Source temperature temperature	120 °C
Desolvation temperature	550 °C
Gas flow	1000 L/h
Cone gas	50 L/h
Collision energy low energy	6 eV
Collision energy High energy ramp	15-45 eV
Drift gas (CCS)	Helium

Table S6: Diffraction data collection and refinement statistics for the *Cb*Tan2 apo structure.

**Accession code**	7Q6Y
**Data collection**	
Wavelength	0.8731
Resolution range	46.45 - 2.22 (2.299 - 2.22)
Space group	$P2_12_12_1$
Unit cell (a,b,c; α,β,γ)	90.675, 95.003, 131.586; 90, 90, 90
Total reflections	748125 (75538)
Unique reflections	56815 (5560)
Multiplicity	13.2 (13.5)
Completeness (%)	99.94 (99.75)
Mean I/sigma(I)	11.93 (1.10)
R-merge (all I+ & I-)	0.196 (2.463)
R-meas (all I+ & I-)	0.204 (2.56)
R-pim (all I+ & I-)	0.05603 (0.6927)
CC1/2	0.998 (0.497)
CC*	1 (0.815)
**Refinement**	
R-work	0.2001 (0.3516)
R-free (5% reflections)	0.2536 (0.3901)
Number of non-hydrogen atoms	7678
macromolecules	7371
ligands	79
solvent	228
Protein residues	929
RMS (bonds)	0.008
RMS (angles)	0.97
Ramachandran favored (%)	94.89
Ramachandran allowed (%)	4.79
Ramachandran outliers (%)	0.33
Rotamer outliers (%)	3.12
Clashscore	5.20
Average B-factor	54.34
macromolecules	54.38
ligands	62.58
solvent	49.97

Statistics for the highest-resolution shell are shown in parentheses.

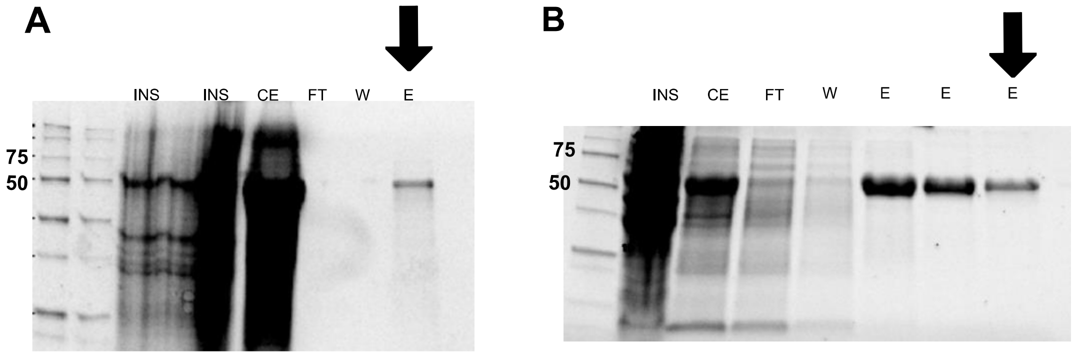

Figure S1. SDS-PAGE analysis of purified *Cb*Tan1 and 2, with the relevant protein ladder markers' molecular weights in kDa. Arrows indicate the lane with protein used for kinetic experiments. Lanes were loaded with insoluble material (INS), crude extract (CE), flow though from IMAC (FT), wash from IMAC (W), and elution from IMAC (E; from multiple aliquots in B). A) *Cb*Tan1 (65 kDa) and B) *Cb*Tan2 (55kDa).

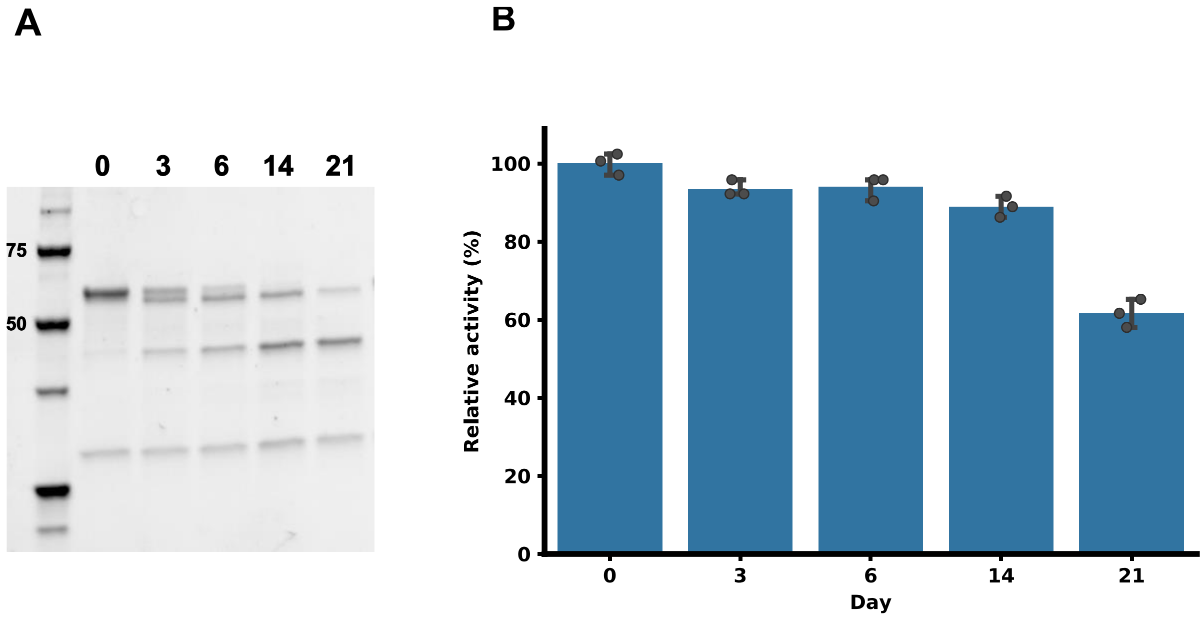

Figure S2. Degradation of *Cb*Tan3 over time (55 kDa). The protein ladder is marked with relevant molecular weights (kDa). A) SDS-PAGE gel loaded with the same volume sample in all wells from samples collected over 4 weeks (days indicated above each lane). B) Relative enzyme activity of *Cb*Tan3 over time using 1 mM methyl gallate and the rhodanine assay. The activity was normalized to tannase activity measured at day 0. The relative activity decrease correlates to the intensity decrease of the 55 kDa band. The individual data points are shown, and also shown as means with standard deviations.

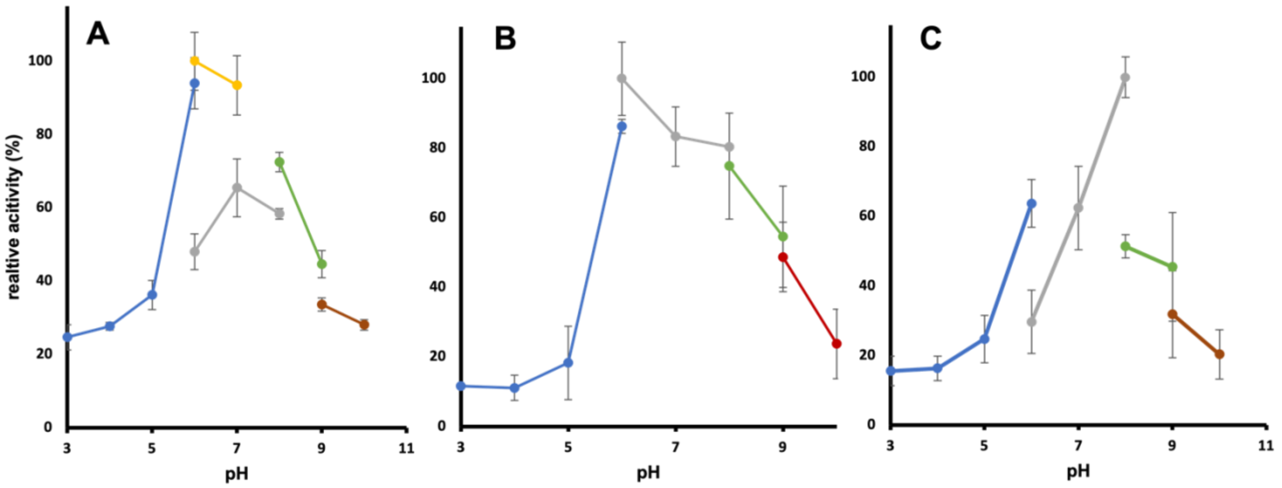

Figure S3. Effect of pH on tannase activity. The enzyme activity is normalized and displayed relative to the highest measured activity. The pH optima for the tannases were determined using the rhodanine assay with 1 mM methyl gallate, varying the 100 mM buffer used, sodium citrate (blue), sodium phosphate (grey), Bis-(2-hydroxyethyl) amino-tris (hydroxymethyl) methane (BIS-TRIS) (yellow), tris(hydroxymethyl)aminomethane (TRIS) (green), N-Cyclohexyl-2-aminoethanesulfonic acid (CHES) (red) A) *Cb*Tan1 with a pH optimum of 6-7 in BIS-TRIS B) *Cb*Tan2 with a pH optimum of 6-7 in sodium phosphate C) *Cb*Tan3 with a pH optimum of 8 in sodium phosphate. The measurements are represented as means and standard deviations from triplicate experiments.

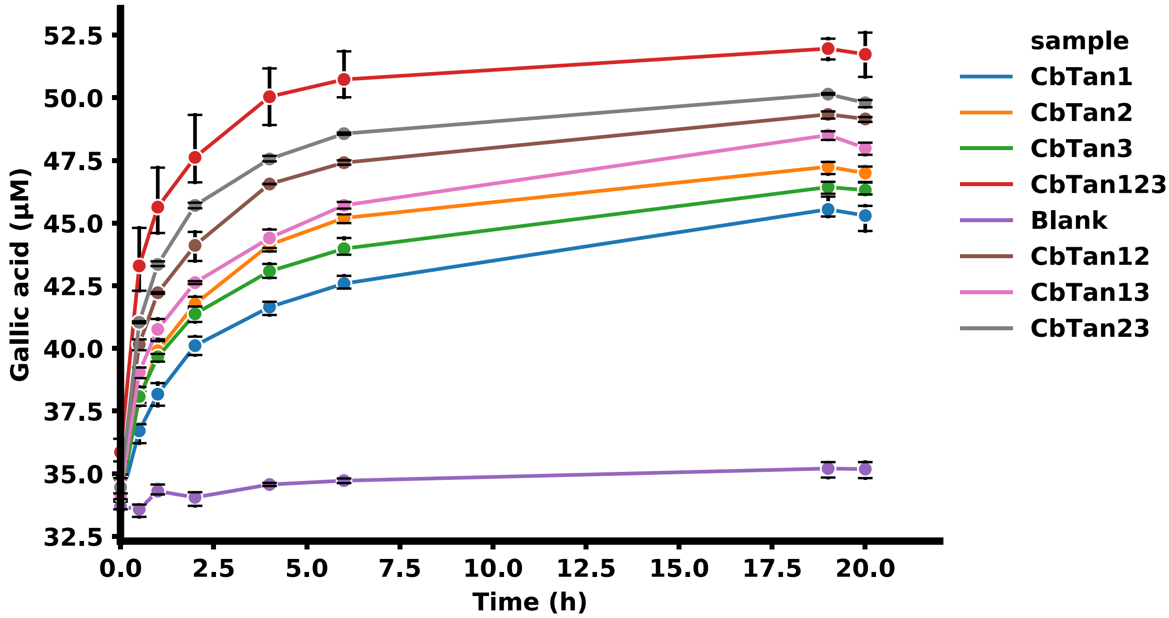

Figure S4. Effect of enzyme treatment of water-extracted oak bark over time. Released gallic acid in the enzymatically treated oak bark, using *Cb*Tan1 (blue), *Cb*Tan2 (orange), *Cb*Tan3 (green), the sample containing all three enzymes *Cb*Tan1-3 (red), *Cb*Tan1+2 (brown), *Cb*Tan1+3 (pink), *Cb*Tan2+3 (grey) compared to a sample without any enzyme (purple). No apparent synergistic effect can be seen in the sample containing all enzymes or the samples containing two enzymes. The measurements are represented as means and standard deviations from triplicate experiments.

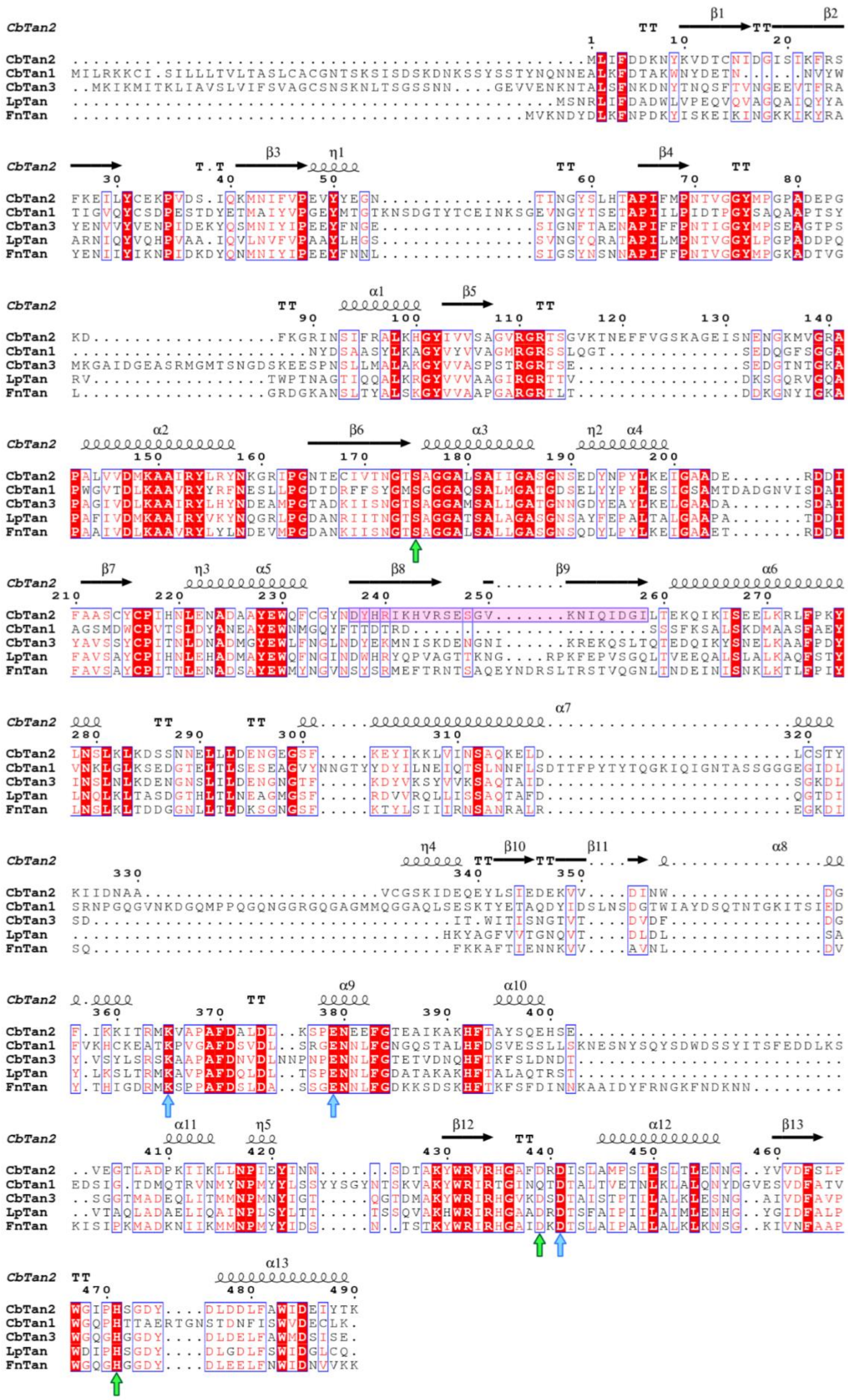
CbTan2
CbTan1
CbTan3
LpTan
FnTan

Figure S5. Sequence-based alignment of selected tannases with available structural information. Shown are the three *Cb*Tan enzymes and the previously characterized FnTan and TanLp. Green arrows show the catalytic triad. Of note is the different catalytic residue between *Cb*Tan1 and the other tannases shown (Q vs D). Blue arrows indicate the gallate binding residues. The pink box highlights the cap domain as determined with the newly solved structure of *Cb*Tan2 (residues 237-258). Of further note is the numerous insert regions unique to each tannase, with no significant homology of these inserts between the tannases. The secondary structural motifs above the sequences represent those from the solved *Cb*Tan2 structure. The alignment was created using ClustalOmega (5) and visualized using ESPript 3 (6).

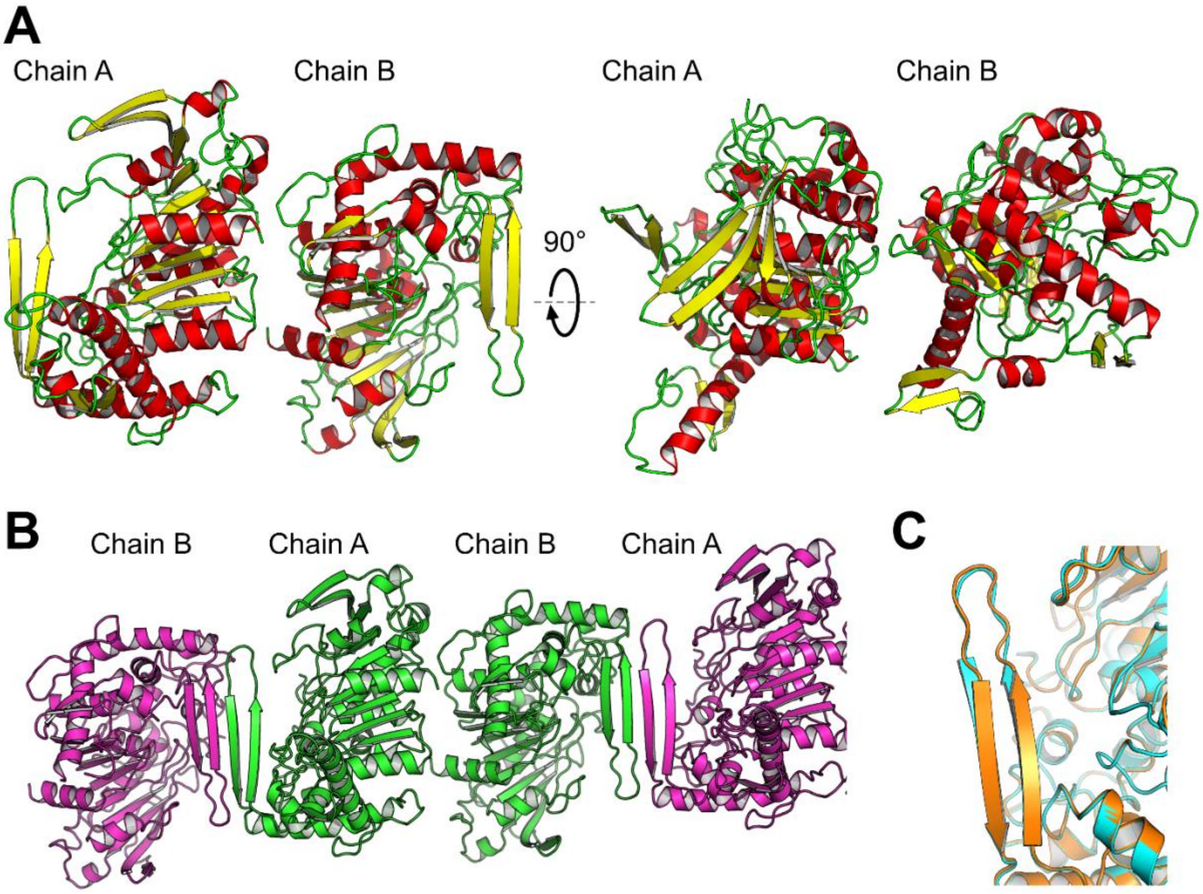

Figure S6: Composition of the asymmetric unit and symmetry contacts of the newly solved *Cb*Tan2. The two chains are labelled Chain A and B. A) Composition of the asymmetric unit. The colours represent secondary structural features, with β-sheets in yellow, α-helices in red and loops in green. B) Crystal contacts between symmetry mates. *Cb*Tan2 is shown in green, and the nearest symmetry neighbors are shown in magenta, highlighting the close contacts between the Chain A lid with the Chain B lid. C) Overlay of the lid domains. Chain A is cyan; Chain B is orange.

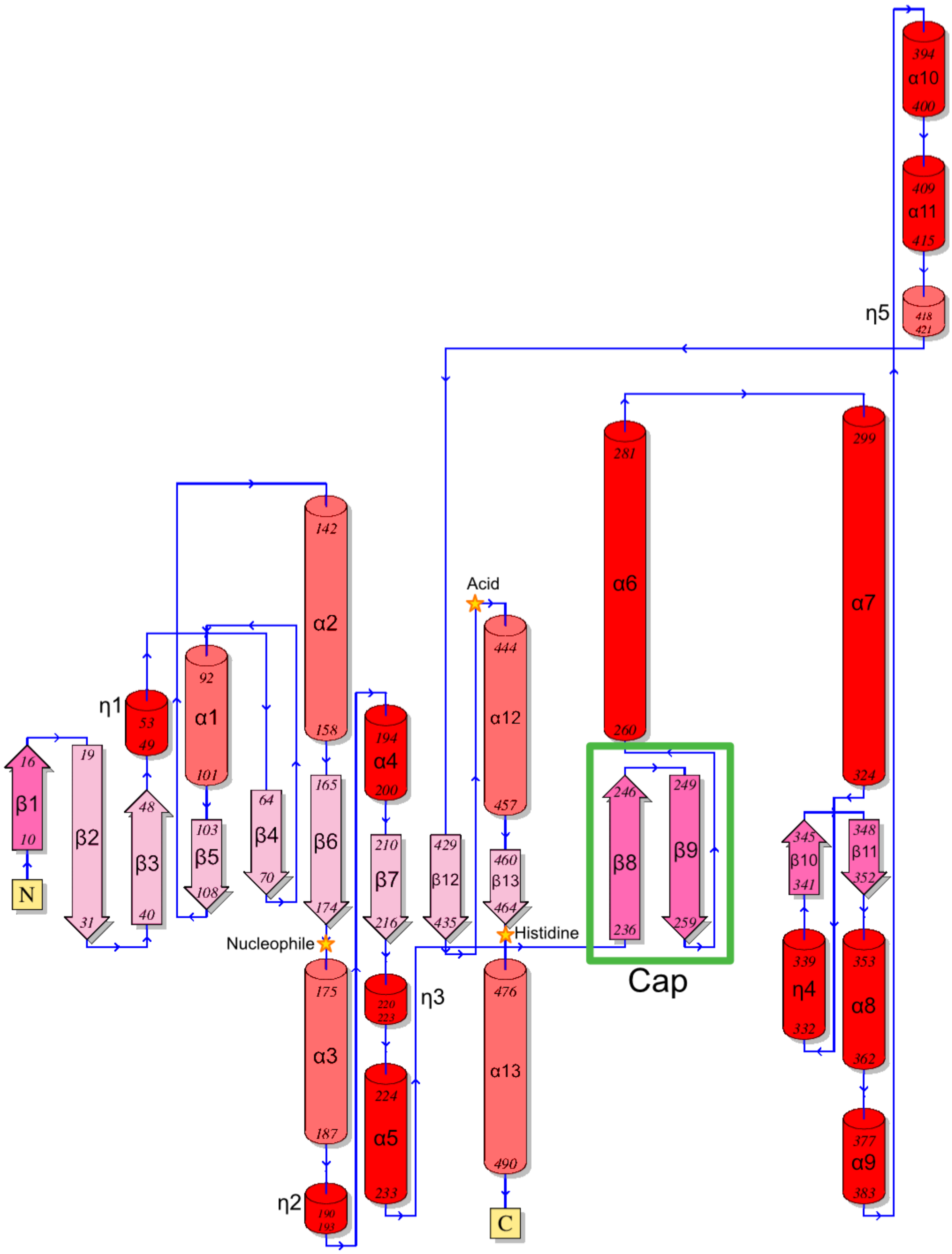

Figure S7. Protein topology map of *Cb*Tan2. The secondary feature numbering is based on Figure S6. The light red helices and light pink sheets denote features present in the conserved α/β hydrolase fold, while the darker red helices and darker pink sheets are inserts unique to *Cb*Tan2. The green box shows the cap domain. Stars show the locations of the catalytic triad, labelled "Acid", "Nucleophile" and "Histidine".

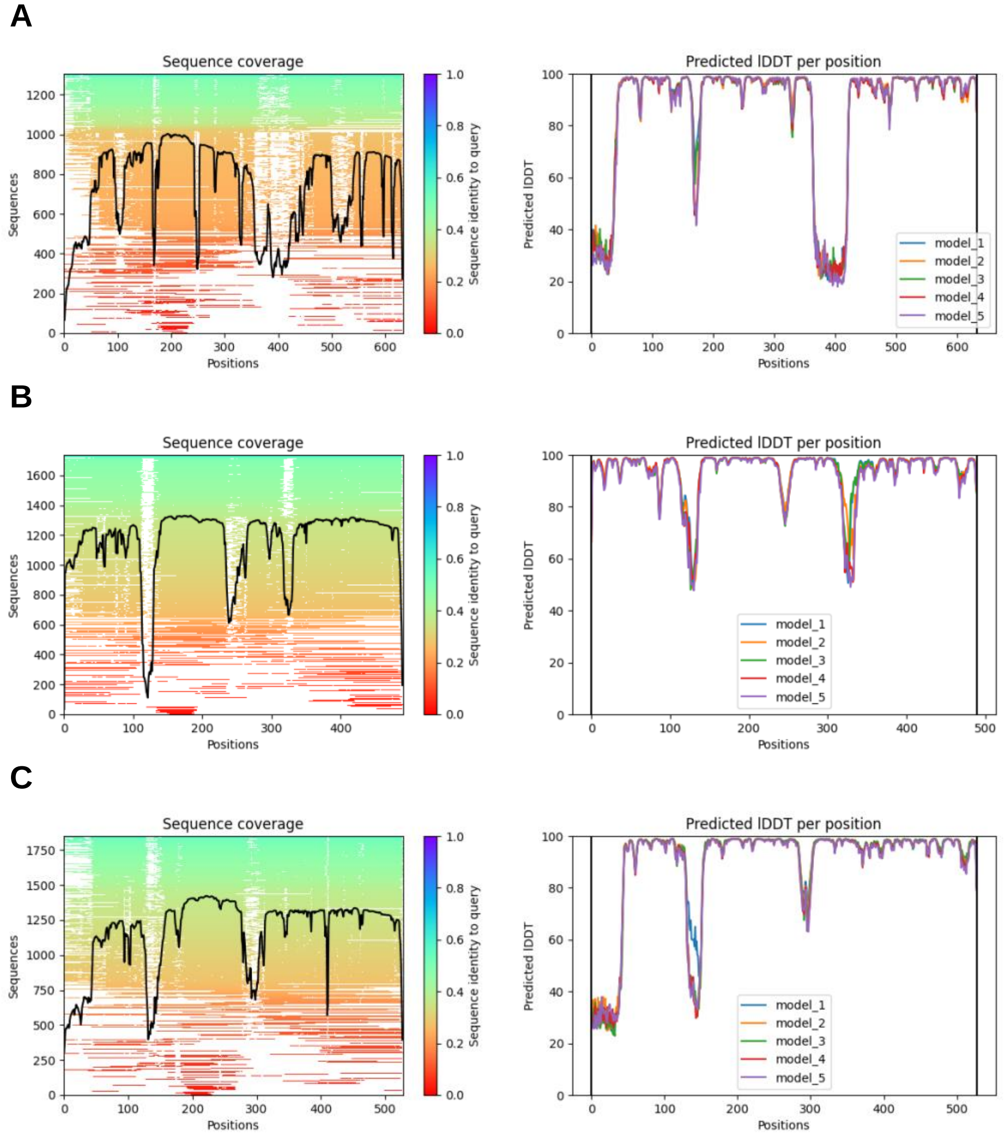

Figure S8. Sequence coverage and prediction scores for each *Cb*Tan model generated using ColabFold. A) *Cb*Tan1, B) *Cb*Tan2, C) *Cb*Tan3. In each, the left plot shows the number of sequences used to predict each residue position, while the right plot shows the predicted pIDDT score per residue position. The prediction score is high (pIDDT close to 100) for the conserved α/β hydrolase fold regions, but less confident for the non-conserved insert regions unique to each *Cb*Tan. In each case, the models are sorted by descending quality from 1 to 5 and model 1 was selected in each case. For each *Cb*Tan, the 5 models aligned with Cα RMSD < 0.18 indicating the predicted structures were convergent on a final model.

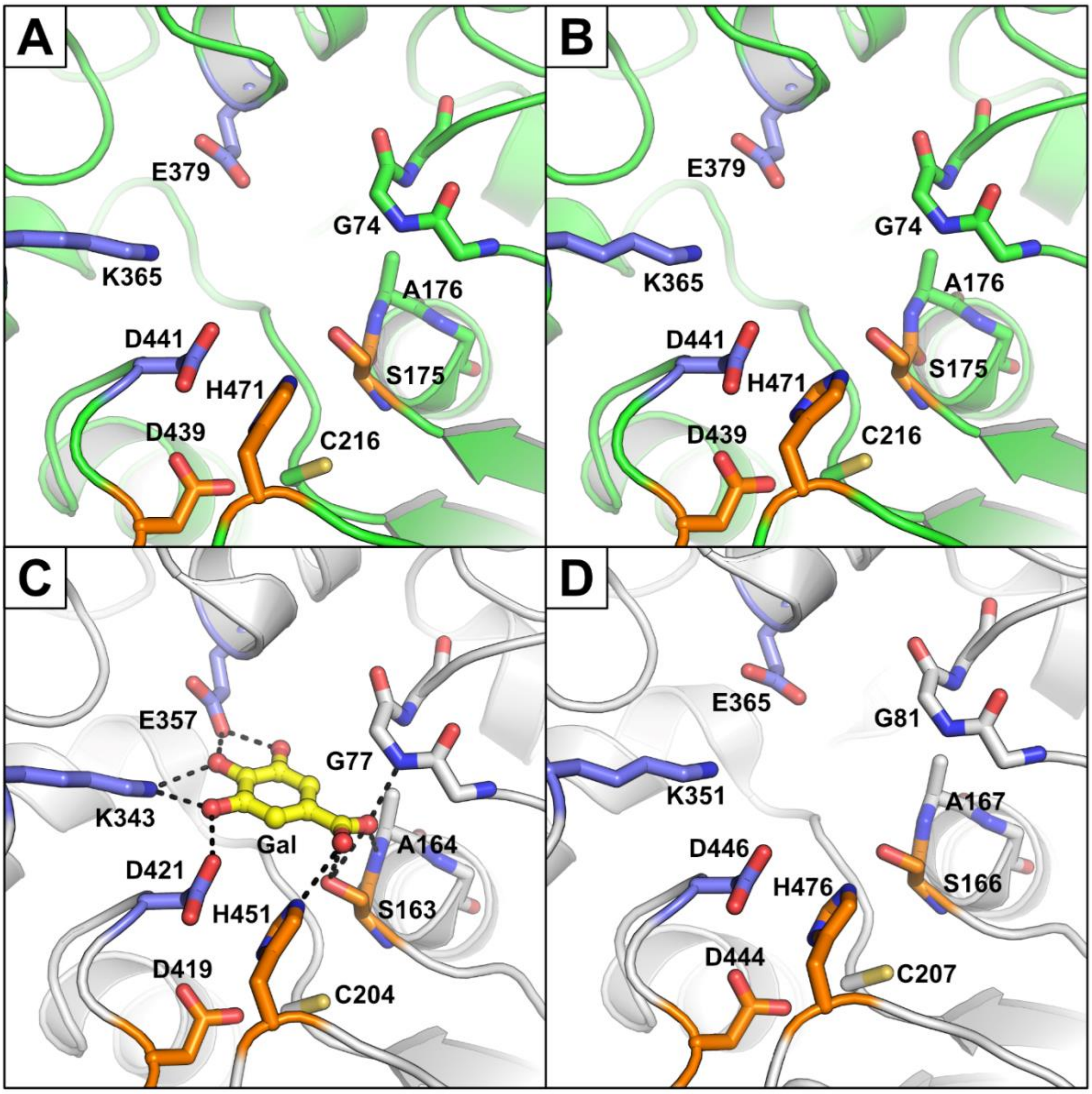

Figure S9: Comparison of active sites of solved bacterial Tannases. A) Chain A of *Cb*Tan2 solved in this study, in green, with catalytic residues in orange and gallate binding residues in purple. B) Chain B of *Cb*Tan2. C) TanLp, with gallate in yellow. Interactions to active site residues and oxyanion hole are shown as dashed lines. D) FnTan. In each, the section of peptide backbone containing the oxyanion hole is labelled, and colored the same as the overall structure.

References

1. Song, X.-C., Canellas, E., Dreolin, N., Nerin, C., and Goshawk, J. (2021) Discovery and Characterization of Phenolic Compounds in Bearberry (Arctostaphylos uva-ursi) Leaves Using Liquid Chromatography–Ion Mobility–High-Resolution Mass Spectrometry. *Journal of Agricultural and Food Chemistry*
2. Venter, P., Causon, T., Pasch, H., and de Villiers, A. (2019) Comprehensive analysis of chestnut tannins by reversed phase and hydrophilic interaction chromatography coupled to ion mobility and high resolution mass spectrometry. *Analytica chimica acta* **1088**, 150-167
3. Colby, S. M., Nuñez, J. R., Hodas, N. O., Corley, C. D., and Renslow, R. R. (2019) Deep learning to generate in silico chemical property libraries and candidate molecules for small molecule identification in complex samples. *Analytical chemistry* **92**, 1720-1729
4. Chernonosov, A. A., Karpova, E. A., and Lyakh, E. M. (2017) Identification of phenolic compounds in Myricaria bracteata leaves by high-performance liquid chromatography with a diode array detector and liquid chromatography with tandem mass spectrometry. *Revista Brasileira de Farmacognosia* **27**, 576-579
5. Madeira, F., Park, Y. M., Lee, J., Buso, N., Gur, T., Madhusoodanan, N., Basutkar, P., Tivey, A. R. N., Potter, S. C., Finn, R. D., and Lopez, R. (2019) The EMBL-EBI search and sequence analysis tools APIs in 2019. *Nucleic acids research* **47**, W636-W641
6. Robert, X., and Gouet, P. (2014) Deciphering key features in protein structures with the new ENDscript server. *Nucleic Acids Research* **42**, W320-W324
