# Supplementary material for: Structural diversity and substrate preferences of three tannase enzymes encoded by the anaerobic bacterium Clostridium butyricum
Source: J Biol Chem. 2022 Feb 21;298(4):101758. doi: 10.1016/j.jbc.2022.101758 (PMC8958541; doi:10.1016/j.jbc.2022.101758)

Supplemental information file 2. Phylogenetic tree of identified putative tannases. Characterized enzymes are named, e.g. TanLp, while the others are shown with their respective IMG accession numbers and locus tags.
Bootstrap values are shown at each branch, from 1000 ultrafast bootstraps in IQ-TREE (see Methods).

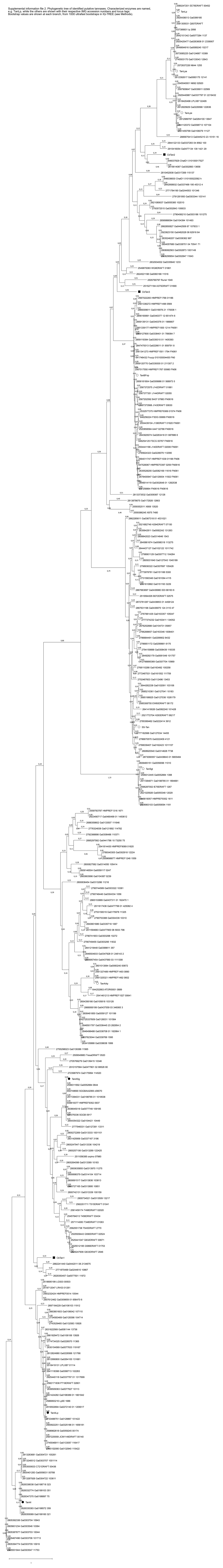

Supplement: Supplementary Information File 2 [file mmc2.pdf]
